# Adipose ABHD6 regulates tolerance to cold and thermogenic programs

**DOI:** 10.1172/jci.insight.140294

**Published:** 2020-12-17

**Authors:** Pegah Poursharifi, Camille Attané, Yves Mugabo, Anfal Al-Mass, Anindya Ghosh, Clémence Schmitt, Shangang Zhao, Julian Guida, Roxane Lussier, Heidi Erb, Isabelle Chenier, Marie-Line Peyot, Erik Joly, Christophe Noll, André C. Carpentier, S.R. Murthy Madiraju, Marc Prentki

**Affiliations:** 1 Departments of Nutrition, Biochemistry, and Molecular Medicine, University of Montreal, and Montreal Diabetes Research Center, Centre de Recherche du Centre Hospitalier de l’Université de Montréal (CRCHUM), Montréal, Québec, Canada.; 2 Institut de Pharmacologie et de Biologie Structurale, Université de Toulouse, CNRS, UPS, Toulouse, France.; 3 Department of Medicine, McGill University, Montréal, Québec, Canada.; 4 Touchstone Diabetes Center, UT Southwestern Medical Center, Dallas, Texas, USA.; 5 Division of Endocrinology, Department of Medicine, Centre de recherche du Centre Hospitalier Universitaire de Sherbrooke, Université de Sherbrooke, Sherbrooke, Québec, Canada.

**Keywords:** Metabolism, Adipose tissue

## Abstract

Enhanced energy expenditure in brown (BAT) and white adipose tissues (WAT) can be therapeutic against metabolic diseases. We examined the thermogenic role of adipose α/β-hydrolase domain 6 (ABHD6), which hydrolyzes monoacylglycerol (MAG), by employing adipose-specific ABHD6-KO mice. Control and KO mice showed similar phenotypes at room temperature and thermoneutral conditions. However, KO mice were resistant to hypothermia, which can be accounted for by the simultaneously increased lipolysis and lipogenesis of the thermogenic glycerolipid/free fatty acid (GL/FFA) cycle in visceral fat, despite unaltered uncoupling protein 1 expression. Upon cold stress, nuclear 2-MAG levels increased in visceral WAT of the KO mice. Evidence is provided that 2-MAG causes activation of PPARα in white adipocytes, leading to elevated expression and activity of GL/FFA cycle enzymes. In the ABHD6-ablated BAT, glucose and oxidative metabolism were elevated upon cold induction, without changes in GL/FFA cycle and lipid turnover. Moreover, response to in vivo β_3_-adrenergic stimulation was comparable between KO and control mice. Our data reveal a MAG/PPARα/GL/FFA cycling metabolic signaling network in visceral adipose tissue, which contributes to cold tolerance, and that adipose ABHD6 is a negative modulator of adaptive thermogenesis.

## Abstract 2

Visceral adipose adipose α/β-hydrolase domain 6 regulates cold adaptation and acts as a brake for heat production via the regulation of thermogenic glycerolipid/free fatty acid cycling.

## Graphical abstract

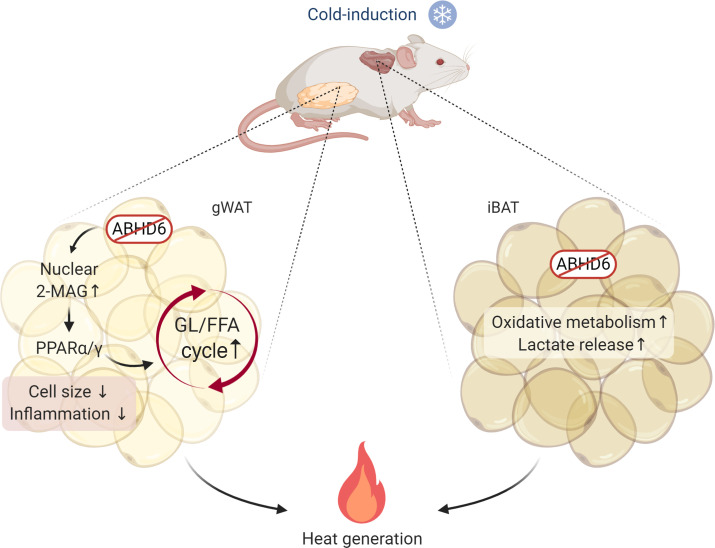

## Introduction

Cold endurance and survival of endotherms depends on endogenous heat production mechanisms involving involuntary muscle contractions, or shivering, and nonshivering thermogenesis to maintain a constant core body temperature (1). Adaptation to nutritional and environmental stresses involves fine-tuning of lipid metabolism and changes in fuel partitioning in fat depots. Traditionally, white adipose tissue (WAT) is considered a key energy reservoir, whereas brown adipose tissue (BAT) contributes to adaptive thermogenesis and body temperature control by dissipating energy (2). Mitochondrial uncoupling protein 1 (UCP1), expressed in high levels in BAT (3), was believed to be the main component of heat-generating machinery (4). However, the thermogenic capacity of WAT is now appreciated. In fact, accumulating evidence suggests the existence of alternative thermogenic mechanisms in WAT that do not require UCP1, via ATP-consuming fuel mobilization processes, mitochondrial dynamics, and more (5–10). The importance of UCP1-independent mechanisms of thermogenesis became evident from the observations that UCP1-KO mice can develop tolerance to hypothermia (6). Also, BAT is either absent or inactive in some mammals such as pigs, and in humans its mass and activity generally decline with age (11). The rediscovery of functional BAT in adult humans (12) has rekindled the interest to combat obesity and metabolic diseases by pharmacological activation of BAT (13). In addition, WAT also has a remarkable capacity to respond to thermogenic stimulations by remodeling into beige adipocytes, possibly contributing to adaptive thermogenesis (14). Considering the large size of WAT depots in the body compared with the BAT, WAT independently of the beiging process may serve as an important tissue for thermogenesis and tolerance to cold. However, much remains to be learned about the pathways and key players implicated in WAT under cold stress.

Among the various stimuli that activate the heat-generating pathways (2, 9), cold exposure is the most powerful trigger to enhance adipose nonshivering thermogenesis that helps in maintaining stable core temperature. The sympathetic nervous system (SNS) transmits cold sensation to fat depots through the release of norepinephrine (4), which initiates thermogenic pathways by activating β-adrenergic receptor/protein kinase A (β-AR/PKA) signaling cascade. This increases expression of UCP1 and PPARγ coactivator 1α (PGC1α), followed by PPARα activation, triggering a thermogenic program in brown and beige adipocytes (15). PKA also enhances lipolysis in fat depots in cold conditions, which is an important contributor to the thermogenesis (4). Furthermore, inhibition of triglyceride (TG) lipolysis in WAT and BAT abolishes cold-induced thermogenesis in rats (16) and in humans (17).

In adipocytes, the TG pool is in a dynamic state, depending on the flux through the lipolysis and lipogenesis arms of the glycerolipid/free fatty acid (GL/FFA) cycle (7). During lipolysis, TG is hydrolyzed sequentially to diacylglycerol (DAG), monoacylglycerol (MAG), and glycerol plus FFA, by adipose triglyceride lipase (ATGL), hormone sensitive lipase (HSL), and monoacylglycerol lipase (MAGL; *Mgll*) and/or α/β-hydrolase domain 6 (ABHD6). MAGL and ABHD6 are sequestered in different subcellular locations, hydrolyzing distinct pools of MAG, and thus likely play distinct physiological roles (18). Despite the high expression level of MAGL in the WAT, it cannot entirely account for the overall stimulated MAG hydrolysis (19). The importance of ABHD6-mediated MAG hydrolysis in cellular signaling became evident from our previous studies, using whole-body and β cell–specific ABHD6-KO mice, that demonstrated a role for 1-MAG, in the regulation of islet β cell insulin secretion (20, 21). Also, we documented that whole-body ABHD6-KO mice are protected from diet-induced obesity, insulin resistance, and hepatic steatosis (22). Although studies on UCP1-independent mechanisms continue to yield novel insights into the energy-dissipating capacity of WAT, which can be of therapeutic value in the treatment of metabolic diseases, the significance of white fat depots in whole-body thermogenesis is still debatable (23, 24). In particular, it is not known if the GL/FFA cycle in WAT contributes to cold-induced thermogenic processes and if adipose tissue ABHD6 plays a regulatory role in this process.

In the current study we examined the contribution of adipose ABHD6 to lipolysis in various fat depots and its thermoregulatory function at thermoneutrality, room temperature (RT), and under cold stress using adipose-specific ABHD6-KO mice. The evidence indicates that deletion of adipose ABHD6 enhances the thermogenic GL/FFA cycle activity in visceral WAT via MAG/PPARα to promote adaptive thermogenesis under cold stress. The results identify ABHD6 as a negative modulator of WAT thermogenesis and implicate the MAG/PPARα/GL/FFA cycle metabolic signaling network as a potentially novel pathway in cold adaptation.

## Results

### Suppression of adipose ABHD6 reduces stimulated lipolysis in 3T3-L1 and primary gonadal adipocytes and basal lipolysis in interscapular BAT explants.

**Figure 1:**
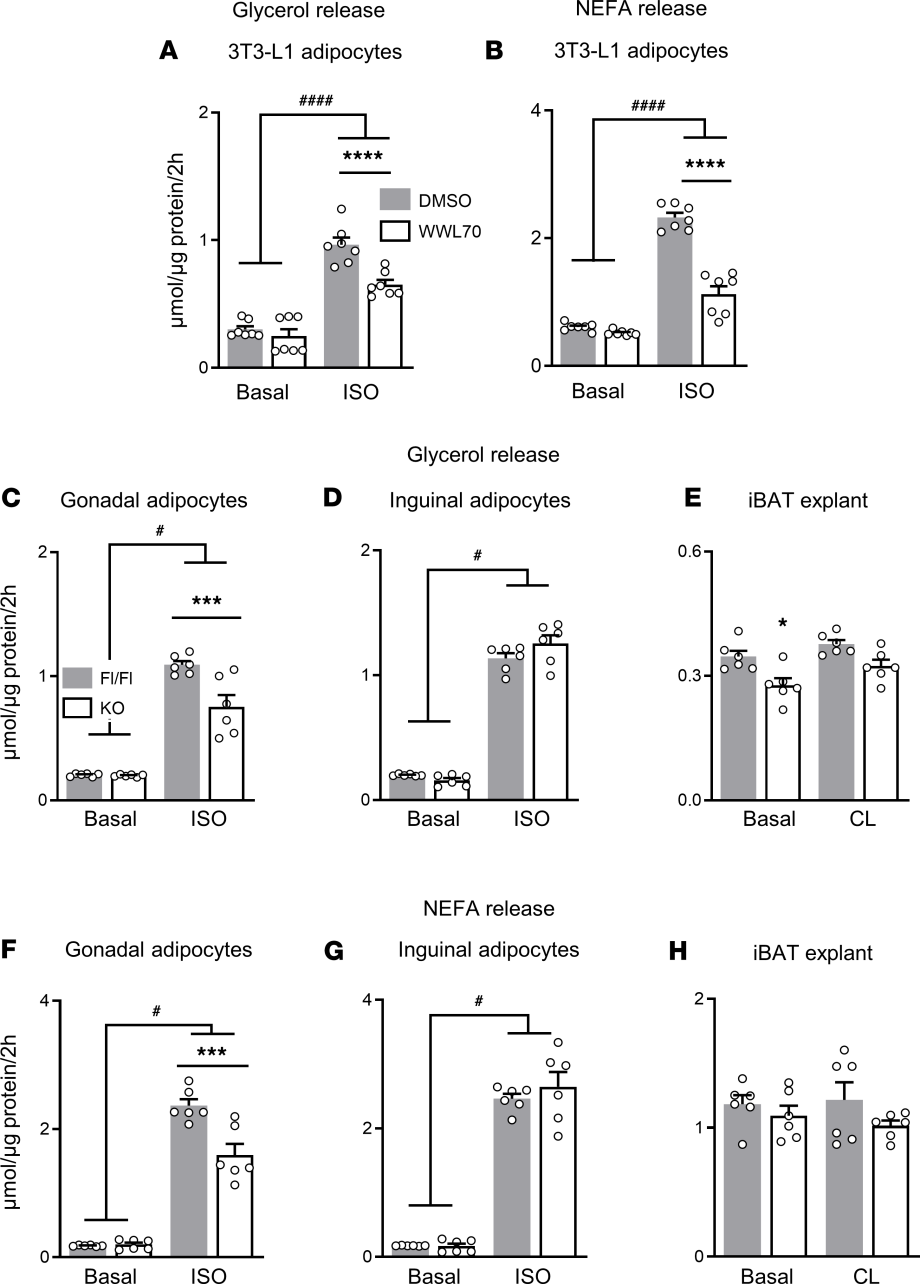
Suppression of adipose ABHD6 reduces stimulated lipolysis in 3T3-L1 and primary gonadal adipocytes and basal lipolysis in intercapsular BAT explants. Adipocytes/tissue explants were stimulated with or without isoproterenol (ISO; 1 μM) or CL316243 (CL; 10 μM), and glycerol and NEFA releases were measured. (**A** and **B**) 3T3-L1 adipocytes (minimum 6 wells/group) ± ABHD6 inhibitor WWL70 (25 μM). (**C**–**H**) Primary white adipocytes and intercapsular BAT (iBAT) explants (6 mice/group). One-way ANOVA and Tukey’s post hoc test. Effects of genotype/inhibitor: *P < 0.05, ***P <0.001, ****P < 0.0001; effects of adrenergic stimulation: ^#^*P* < 0.05, ^####^*P* < 0.0001.

Using global ABHD6-KO mice, we have previously identified ABHD6 as a regulator of systemic energy balance (22), though the adipocyte-intrinsic role of ABHD6 remained uncertain. In order to clarify the metabolic role of ABHD6 in fat cells, we first evaluated the effect of ABHD6 inhibition in vitro on 3T3-L1 adipocyte lipolysis under basal and isoproterenol-stimulated (ISO-stimulated) conditions. ABHD6 inhibition with WWL70 resulted in decreased release of ISO-stimulated glycerol and nonesterified fatty acids (NEFAs) (*P* < 0.0001) but not basal lipolysis (Figure 1, A and B).

Next, we generated inducible adipose-specific ABHD6-KO (AT-ABHD6–KO) mice on C57BL/6N genetic background (25). Male 8-week-old AT-ABHD6–KO mice and their littermate controls (*Abhd6*^fl/fl^ [Fl/Fl] and *Adipoq-Cre* [Cre]) were treated with tamoxifen (TMX) and were kept on a normal diet. Deletion of ABHD6 in fat pads was ascertained 2 weeks after TMX treatment (Supplemental Figure 1, A–C, E and F; supplemental material available online with this article; https://doi.org/10.1172/jci.insight.140294DS1). Ex vivo lipolysis in mature adipocytes was found to be unchanged under basal conditions, but ISO-stimulated lipolysis was reduced in gonadal adipocytes from KO mice (Figure 1, C and F). Basal and ISO-stimulated lipolysis was similar between KO and Fl/Fl mice in inguinal adipocytes (Figure 1, D and G). On the other hand, basal glycerol from iBAT explants showed a modest reduction due to ABHD6 ablation (Figure 1E), without any changes in NEFA release (Figure 1H). The data show that ABHD6 substantially contributed to overall lipolysis (MAG hydrolysis) in 3T3-L1 and primary gonadal adipocytes and suggest a depot-specific role for ABHD6 because it does not contribute to subcutaneous WAT lipolysis.

### Characterization of AT-ABHD6–KO mice at RT.

Chow diet–fed AT-ABHD6–KO mice did not differ in their body weight, food intake, as well as fat and lean mass from their controls (Supplemental Figure 1, G–I). Oral glucose tolerance test (OGTT) showed no differences between AT-ABHD6–KO, Cre, and Fl/Fl control mice in their glycemia or insulinemia (Supplemental Figure 1, J and K). However, AT-ABHD6–KO mice displayed better insulin sensitivity (*P* < 0.05) in insulin tolerance test (ITT) (Supplemental Figure 1L). Taken together, under standard diet and RT conditions, deletion of adipose ABHD6 in adult mice increases whole-body insulin sensitivity but has no effect on overall fat mass as well as glucose and energy homeostasis.

### AT-ABHD6–KO mice show elevated energy expenditure under cold and are resistant to cold-induced hypothermia.

**Figure 2:**
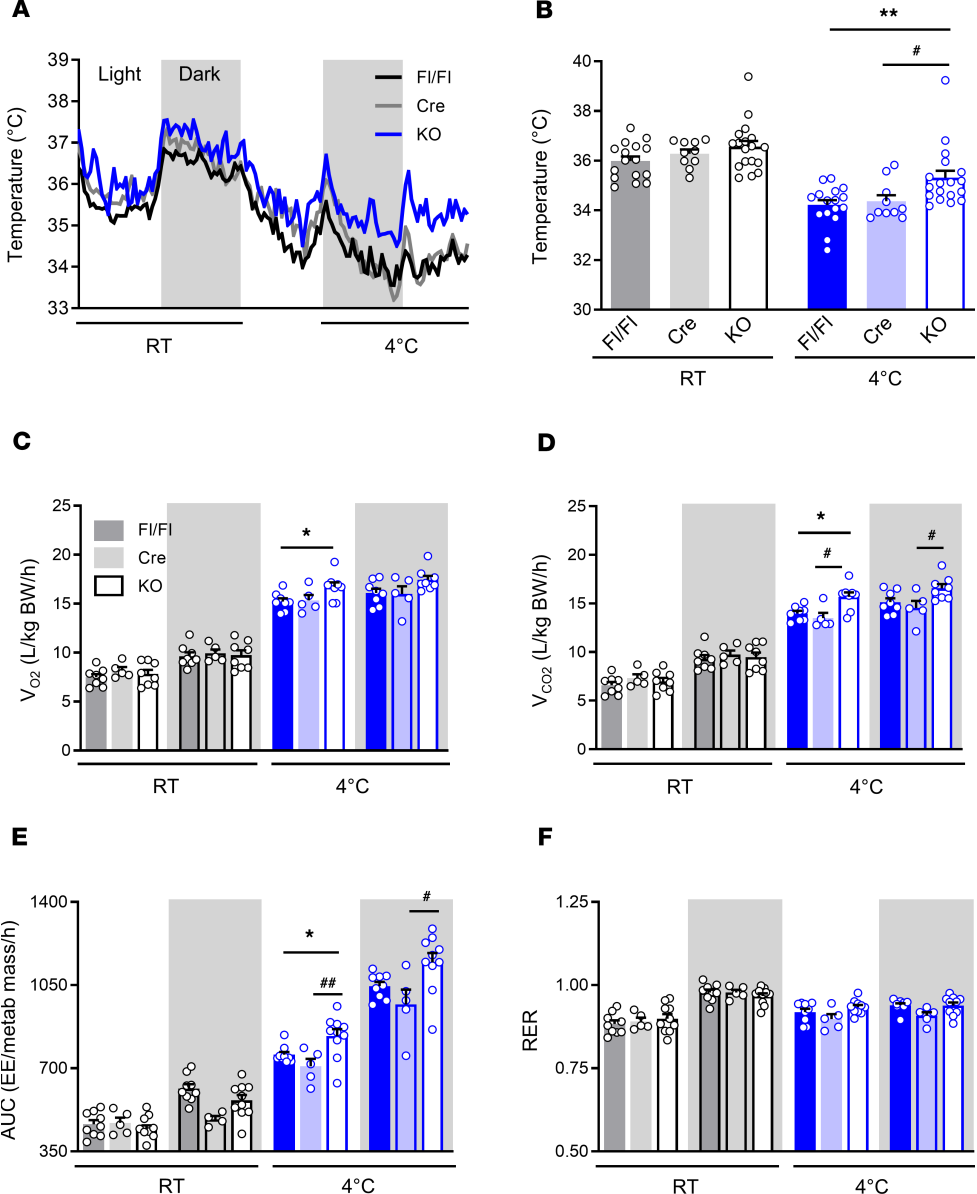
AT-ABHD6–KO mice show elevated energy expenditure under cold and are resistant to cold-induced hypothermia. (**A**) Core body temperature. (**B**) Average core body temperature over 24 hours (light + dark cycles). (**C**) Volume O_2_ (V_O2_). (**D**) Volume CO_2_ (V_CO2_). (**E**) Energy expenditure (EE; kcal/kg metabolic mass/h). (**F**) Respiratory exchange ratio (RER). *n* = 5–9 mice/group; 1-way ANOVA and Tukey’s post hoc test; Fl/Fl vs. KO: *P < 0.05, **P < 0.01; Cre vs. KO: ^#^*P* < 0.05, ^##^*P* < 0.01.

We further examined whether adipose ABHD6 displays a regulatory role in energy balance and adaptive thermogenesis that is autonomous to mature adipocytes, under cold-stress conditions. Control (Fl/Fl and Cre) and AT-ABHD6–KO mice were exposed to 4°C for 3 hours or 24 hours. While there was no difference in body weight and glycemia between KO and controls in all conditions (Supplemental Figure 2, A and B), rectal temperature was maintained more stably at a higher level in AT-ABHD6–KO mice than in controls, during the 3 hours cold exposure and after 24 hours cold exposure (Supplemental Figure 2, C and D). In order to continuously collect various metabolic parameters in a temperature-controlled environment, Fl/Fl, Cre, and KO mice were implanted with intraperitoneal temperature probes and were individually housed in metabolic cages (Comprehensive Lab Animal Monitoring System, CLAMS). Following 24 hours acclimatization at RT, respiration, food intake, core body temperature, and locomotor activity were recorded at RT for another 24 hours. This was followed by gradual decrease of cage temperature to 4°C over a 12-hour period, and metabolic parameters were then recorded at 4°C for 24 hours. When housed at RT, KO and control mice exhibited a similar and stable core body temperature. But under cold conditions, core body temperature dropped to a much lower level in control mice than in the KO mice (Figure 2, A and B). AT-ABHD6–KO mice displayed significantly higher oxygen consumption in light cycle and carbon dioxide production and energy expenditure (EE) in both light and dark cycles at 4°C (Figure 2, C–E), compared with the controls. There were no differences between the groups, in respiratory exchange ratio (Figure 2F), physical activity, and food consumption (Supplemental Figure 2, E and F). To eliminate RT-related cold stress, in another set of experiments, the CLAMS temperature was adjusted to 30°C for 5 consecutive days, and measurements were performed during the last 24 hours of the protocol. All the metabolic parameters were comparable between Fl/Fl and KO mice under thermoneutrality (Supplemental Figure 2, G and H), similar to the RT condition. Although it was previously reported that RT increases the basal metabolic rate in rodents (26), the intensity of the cold stress under 22°C appears to be insufficient to stimulate significant metabolic changes in the absence of ABHD6, especially given that the mice used in this study were born and housed under RT and adapted to their environmental condition.

The in vivo studies using AT-ABHD6–KO mice generated by adipoq-Cre–driven ABHD6 deletion helped in understanding the overall significance of adipose ABHD6 in the control of cold-induced thermogenesis. However, as this model cannot differentiate the role of individual fat depots for in vivo measurements, we performed several ex vivo experiments to assess and better understand the effect of depot-specific ABHD6 deletion.

### Glucose and oxidative metabolism are enhanced in iBAT from AT-ABHD6–KO mice under cold exposure.

**Figure 3:**
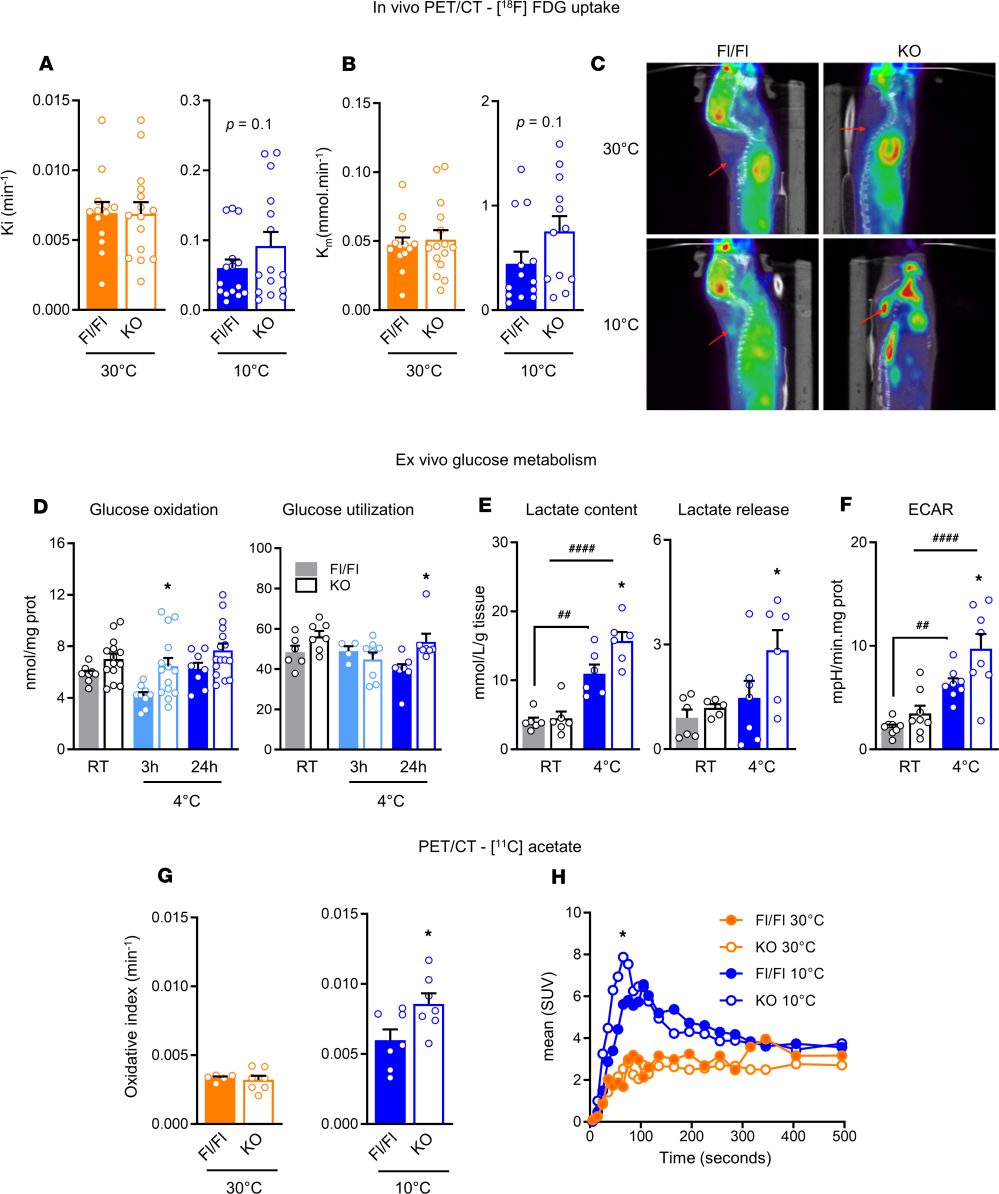
Glucose and oxidative metabolism are enhanced in iBAT from AT-ABHD6–KO mice under cold exposure. (**A**) Fractional (Ki) [^18^F]-FDG uptake, determined by PET/CT. (**B**) Net (Km) [^18^F]-FDG uptake, determined by PET/CT. (**C**) Representative sagittal view of PET/CT images following 10 MBq [^18^F]-FDG injection. The red arrows indicate iBAT localization. (**D**) Ex vivo glucose oxidation (^14^C-glucose) and utilization (^3^H-glucose). (**E**) Tissue lactate content and ex vivo lactate release from tissue explants. (**F**) Extracellular acidification rate (ECAR) on tissue explants. (**G**) Oxidative metabolism index, analyzed by [^11^C]-acetate PET tracer (*P* = 0.03). (**H**) Mean standard uptake value (SUV) of [^11^C]-acetate over the first 500 seconds of acquisition after tracer injection (*P* = 0.01). (**A** and **B**) *n* = 15 mice/group; (**D**–**H**) 5–16 mice/group. (**A**, **B**, and **G**) Student’s *t* test; (**D**–**F**) 1-way ANOVA and Tukey’s post hoc test; (**H**) 2-way ANOVA and Tukey’s post hoc test. The effects of genotype: *P < 0.05; the effects of cold induction: ^##^*P* < 0.01, ^####^*P* < 0.0001.

Because adipose ABHD6 deletion potentiates adaptive thermogenesis under cold stress, we investigated the contribution of the iBAT fuel partitioning and metabolic responses. Glucose uptake was measured in vivo, using [^18^F]-fluorodeoxyglucose ([^18^F]-FDG), by μPET/CT, after exposing the mice (KO and Fl/Fl) at 30°C and 10°C for 48 hours. Cold exposure (10°C) markedly induced the fractional (Ki) and net uptake (Km) of [^18^F]-FDG in both KO and control mice, compared with thermoneutrality (30°C) (Figure 3, A and B). Although large variation observed in [^18^F]-FDG data among animals in both groups under cold and thermoneutral conditions, glucose uptake showed a trend toward increased levels in KO versus Fl/Fl mice under cold (*P* = 0.1) (Figure 3, A and B). Notably, the changes were more evident based on the PET/CT images, which demonstrated an intense focal uptake of [^18^F]-FDG in iBAT of AT-ABHD6–KO mice in response to cold, compared with the control (Figure 3C). Glucose oxidation (after 3 hours cold induction) and glucose utilization (after 24 hours cold induction), measured ex vivo, were enhanced in iBAT from KO versus Fl/Fl mice (*P* < 0.05; Figure 3D). The tissue content and extracellular release of lactate, an indicator of glycolysis, were also significantly elevated by 2-fold in iBAT from KO mice (*P* < 0.05; Figure 3E). Also, the extracellular acidification rate (ECAR), an index of glycolysis, measured using the Seahorse system, was elevated in KO mice after cold exposure (*P* < 0.05; Figure 3F).

PET/CT studies revealed no changes in the Ki and Km of [^18^F]-FDG in visceral perigonadal WAT (gWAT) (Supplemental Figure 3, A and B) and subcutaneous inguinal WAT (iWAT) (data not shown) in the KO mice, compared with controls, regardless of temperature conditions. This is in agreement with gWAT ex vivo glucose metabolism, showing no difference between KO and Fl/Fl mice (Supplemental Figure 3C). However, insulin-stimulated ex vivo glucose uptake in gWAT was significantly elevated in cold-exposed KO mice, compared with controls (Supplemental Figure 3D), and this was in line with the increased glucose transporter type 4 (*Glut4*) expression in gWAT from cold-induced KO mice (Supplemental Figure 3E).

The PET analysis using [^11^C]-acetate, to measure iBAT mitochondrial oxidative capacity in vivo (16), indicated that even though iBAT oxidative metabolism was induced by cold in both KO and Fl/Fl mice, the increase was significantly higher (*P* < 0.05) in AT-ABHD6–KO mice (Figure 3G). Accordingly, the [^11^C]-acetate uptake kinetics curve (Figure 3H) showed that iBAT from KO mice exhibited higher uptake of [^11^C]-acetate in response to cold (*P* < 0.05). Next, we investigated if there are any changes in the mitochondrial biogenesis and function in iBAT. Measurement of mitochondrial respiratory complexes in iBAT showed that the expression of different subunits from complexes I–V was similar in AT-ABHD6–KO and Fl/Fl mice (Supplemental Figure 3F). In addition, isolated iBAT mitochondria were examined for substrate-driven respiration. The oxygen consumption rate (OCR) in the iBAT mitochondria in response to pyruvate/malate (state 2), ADP (state 3), and trifluoromethoxy carbonylcyanide phenylhydrazone (FCCP) (maximal) were comparable between the groups at RT; nevertheless, all these parameters were elevated in both KO and Fl/Fl mice in response to cold stress (Supplemental Figure 3G). Finally, no differences were observed between KO and Fl/Fl mice in the in vivo oxidative activity of gWAT and iWAT, measured using [^11^C]-acetate (data not shown). Thus, the results show that adipose ABHD6 deletion results in enhanced glucose uptake, glycolysis, and oxidative metabolism in iBAT without apparent difference in the white fat depots.

### AT-ABHD6–KO mice show induction of cold-adaptive thermogenesis mechanisms in gWAT distinct from enhanced adipose UCP1 expression.

**Figure 4:**
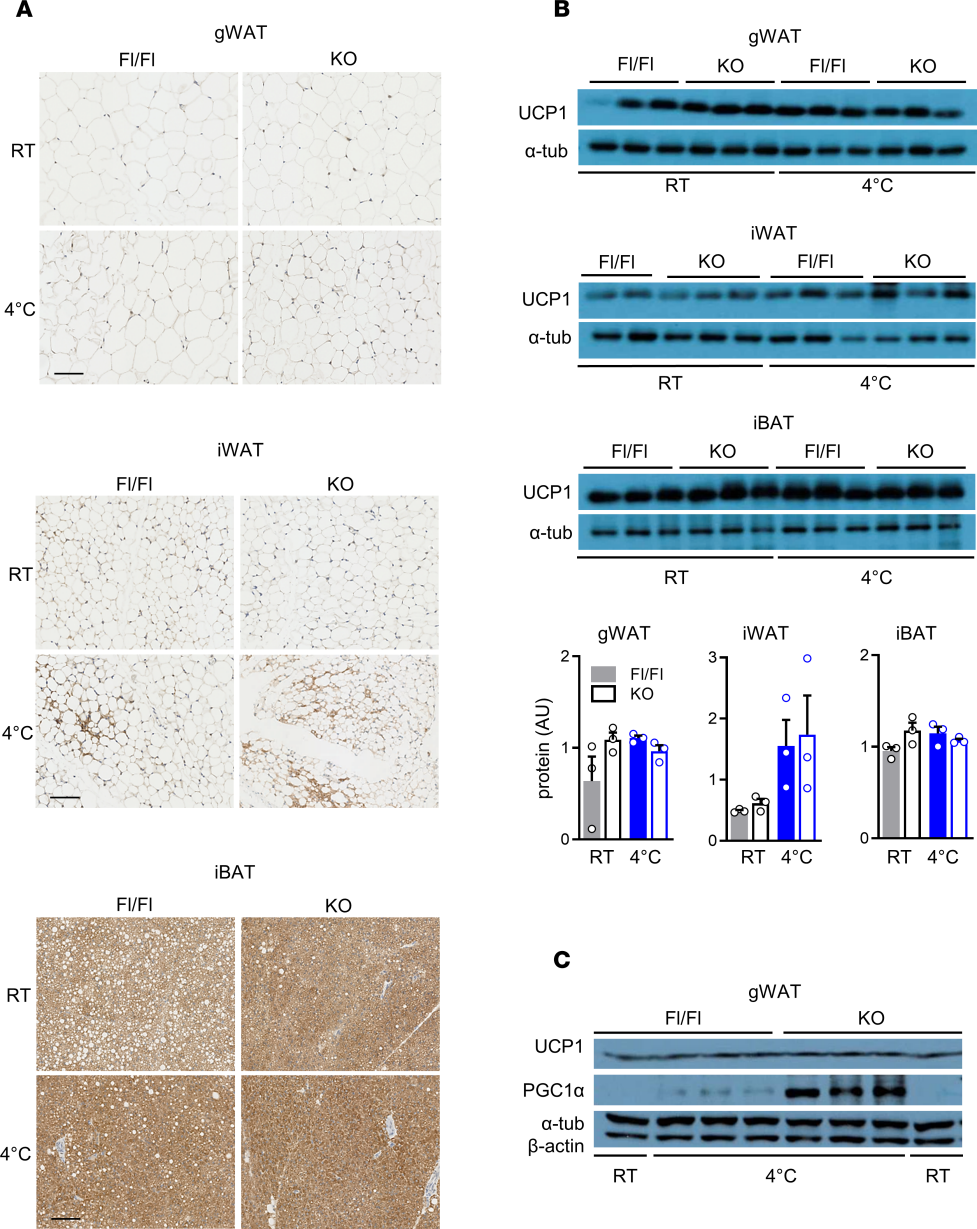
Adipose-specific deletion of ABHD6 does not alter UCP1 expression. (**A**) UCP1 immunostaining. Scale bar: 100 μm. (**B**) UCP1 protein level. (**C**) UCP1 and PGC1α protein levels.

**Figure 5:**
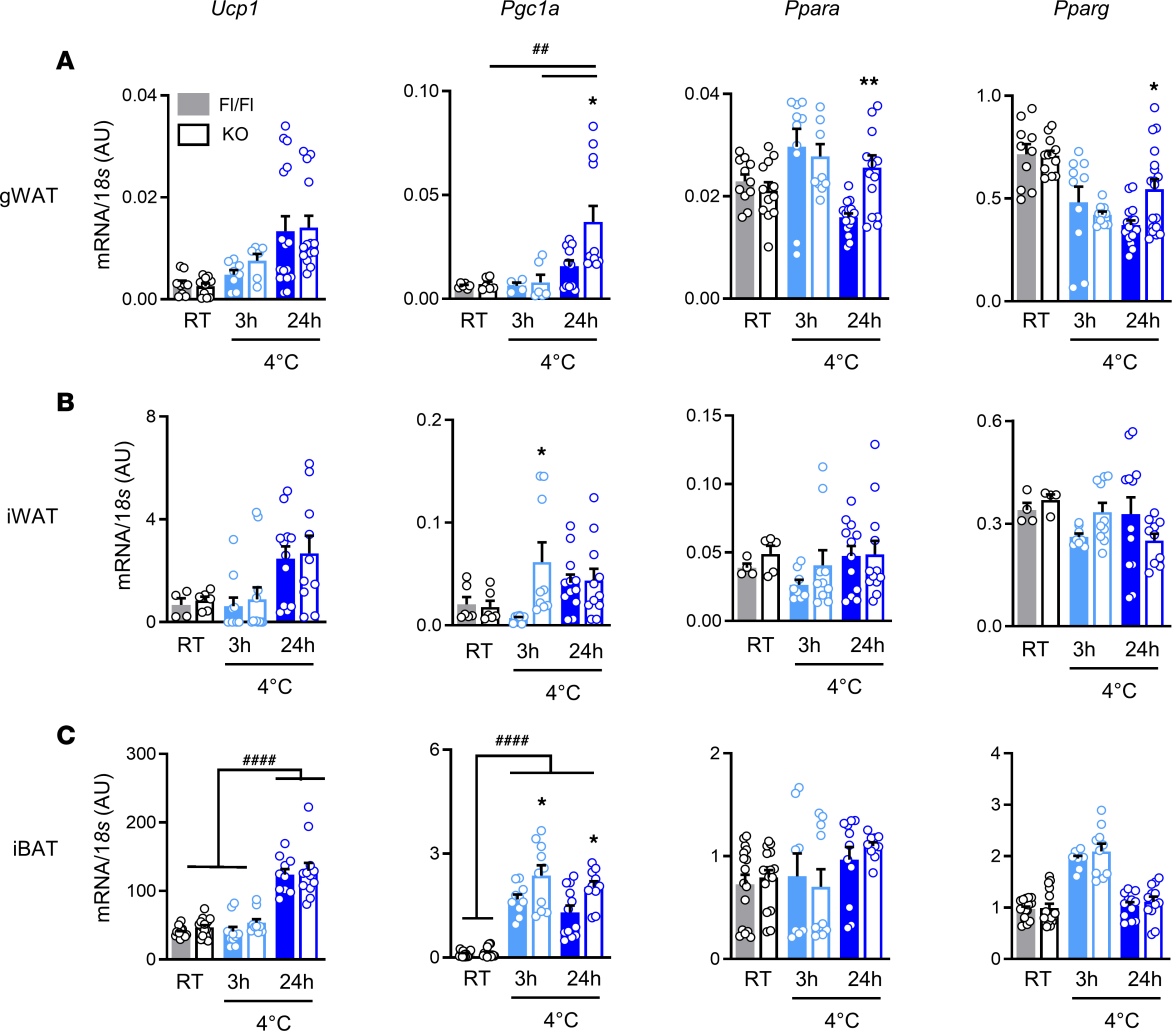
AT-ABHD6–KO mice show induction of cold-adaptive thermogenesis mechanisms in gWAT distinct from enhanced adipose UCP1 expression. (**A**–**C**) *Ucp1*, *Pgc1a*, *Ppara*, and *Pparg* mRNA expression. *n* = 6–18 mice/group; 1-way ANOVA and Tukey’s post hoc test. The effects of genotype: *P < 0.05, **P < 0.01; the effects of cold induction: ^##^*P* < 0.01, ^####^*P* < 0.0001.

Adaptive thermogenesis is generally considered to involve the classical UCP1-dependent mechanisms of BAT activation and WAT browning (3, 4). Besides, our earlier work in whole-body ABHD6-KO mice indicated a potential thermogenic role for UCP1, which was upregulated in all the adipose tissues of KO mice (22). In order to examine the role of UCP1 in the enhanced adaptive thermogenesis seen in the AT-ABHD6–KO mice, we measured UCP1 mRNA and protein levels in fat tissues from animals kept at 30°C or RT and in cold-challenged mice (4°C). Because 3 hours of cold exposure is reported to be sufficient to promote BAT UCP1-dependent metabolism (27), in addition to 24 hours of cold induction, a group of mice were kept 3 hours at 4°C for assessing the effects of acute cold induction. As expected, UCP1 expression was upregulated in fat depots following cold stress in both control and KO mice, in agreement with the known function of UCP1 in adaptive thermogenesis (Figure 4, A and B, and Figure 5, A–C). Our data indicated that cold-mediated adipose UCP1 induction was more prominent after 24 hours versus 3 hours of exposure in both genotypes (Figure 5, A–C). In support of these results, previous reports have also shown that longer duration of cold is essential for restoration of total adipose thermogenic capacity (28). Immunohistochemistry results indicated slightly higher UCP1 protein expression in ABHD6-deficient iBAT at RT (Figure 4A); however, no significant differences were found after 3 hours or 24 hours of cold induction in either mRNA or protein levels in all 3 fat tissues (Figure 4, A–C, and Figure 5, A–C). To eliminate the possible thermogenic activity of adipose depots under RT, we also assessed the thermogenic markers in mice housed at thermoneutrality (30°C) for 5 days. Under thermoneutral conditions UCP1 expression was much lower in iBAT and gWAT, and there were no differences between Fl/Fl and KO groups (Supplemental Figure 4, A and B).

We further analyzed the expression of other genes (*Pgc1a*, *Ppara*, and *Pparg*) known to be involved in WAT browning, BAT activation, and thermogenesis (29). Similar to *Ucp1*, *Pgc1a* was also significantly downregulated at 30°C, and there were no differences between KO and Fl/Fl mice (Supplemental Figure 4, A and B). However, in response to cold exposure, PGC1α protein expression was upregulated markedly in the gWAT from KO mice (Figure 4C). *Pgc1a* mRNA level was elevated under cold in all 3 fat depots of both genotypes, but AT-ABHD6–KO mice exhibited significantly higher levels (3 hours and 24 hours of cold induction), compared with the Fl/Fl mice (Figure 5, A–C). PPARs were similarly expressed in the gWAT of KO and Fl/Fl mice under both 30°C and 22°C conditions (Figure 5A and Supplemental Figure 4, A and B). Expression of both *Ppara* and *Pparg* was decreased in gWAT of Fl/Fl mice upon 24 hours cold stress, whereas no such decrease was seen in the KO mice (Figure 5A). Thus, in concert with PGC1α, after 24 hours cold exposure, there was a significantly elevated level of *Ppara* and *Pparg* mRNA in the gWAT of AT-ABHD6–KO versus Fl/Fl mice (Figure 5A). No change was observed in iWAT and iBAT’s expression of PPARs between AT-ABHD6–KO and Fl/Fl under any temperature condition (Figure 5, B and C, and Supplemental Figure 4, A and B). Thus, the above results show that the increased whole-body thermogenic response of the AT-ABHD6–KO mice to cold is associated with elevated levels of PGC1α, PPARα, and PPARγ in gWAT, but without any concomitant changes in UCP1 level, compared with Fl/Fl mice.

### Smaller gWAT adipocytes and iBAT lipid droplets in cold-exposed AT-ABHD6–KO mice.

In response to hypothermia, adipose tissue is rapidly remodeled with reduction of adipocyte size and lipid droplets. Histological analysis of adipose tissues from Fl/Fl and KO mice showed that cold exposure per se increased the proportion of smaller sized adipocytes in gWAT and of smaller sized lipid droplets in iBAT, with no apparent differences in iWAT (Supplemental Figure 4, C–E). Quantitation of images of H&E-stained tissue sections indicated a greater frequency of smaller adipocytes in gWAT (but not in iWAT) and lipid droplets in iBAT of cold-exposed KO mice versus control (Supplemental Figure 4, F–H), which corresponded to significantly smaller average adipocyte area in gWAT, as well as diminished average lipid droplet size in iBAT (Supplemental Figure 4I). There was a reduction in the size of adipocytes in the iWAT of KO mice only at RT (Supplemental Figure 4I). Following 24 hours cold exposure, there was a decrease in the weights of gWAT, iWAT, and iBAT depots in both genotypes (Supplemental Figure 4J).

### AT-ABHD6–KO mice show enhanced antiinflammatory markers in gWAT.

White adipocyte size is a strong determinant of lipolysis responsiveness, and gWAT adipocyte size, in particular, is known to positively associate with insulin resistance and inflammation (30). The reduced gonadal adipocyte size in KO mice is in line with the changes in inflammatory markers and insulin sensitivity in these mice. Antiinflammatory marker *Arg1* expression was induced in the gWAT from KO mice at RT (Supplemental Figure 4K). After 24 hours cold induction, *Arg1*, *Il10*, and *Adipoq* mRNA levels were significantly higher in the ABHD6-deficient gWAT versus Fl/Fl mice (Supplemental Figure 4, K and L). Besides, the cold-induced increase in adiponectin receptor-2 expression in gWAT was much higher in AT-ABHD6–KO mice than in Fl/Fl mice (Supplemental Figure 4L). In the iBAT, the mRNA expression of *Il10*, *Il4*, and *Arg1* was almost undetectable (data not shown).

### Increased cold-stimulated activity of the lipolytic arm of the GL/FFA cycle in gWAT of AT-ABHD6–KO mice.

GL/FFA cycle has been proposed as a heat-producing and metabolic signal-generating pathway in many cell types (7, 10, 31). Generally, WAT and BAT respond differently under stimulated conditions (32). In WAT there is enhanced lipolytic release of FFAs, which are supplied to BAT for oxidation and heat production and to other energy-demanding organs (33). Because deletion of adipose ABHD6, which besides MAGL catalyzes the last step of the lipolysis arm of the GL/FFA cycle, led to higher energy expenditure and better cold tolerance, we examined the thermogenesis-regulatory role of ABHD6 and how it is related to the GL/FFA cycle in WAT and/or BAT. We first assessed whether the lipolytic arm of this cycle is enhanced in various adipose depots in association with the expression and activity of the lipolytic enzymes.

**Figure 6:**
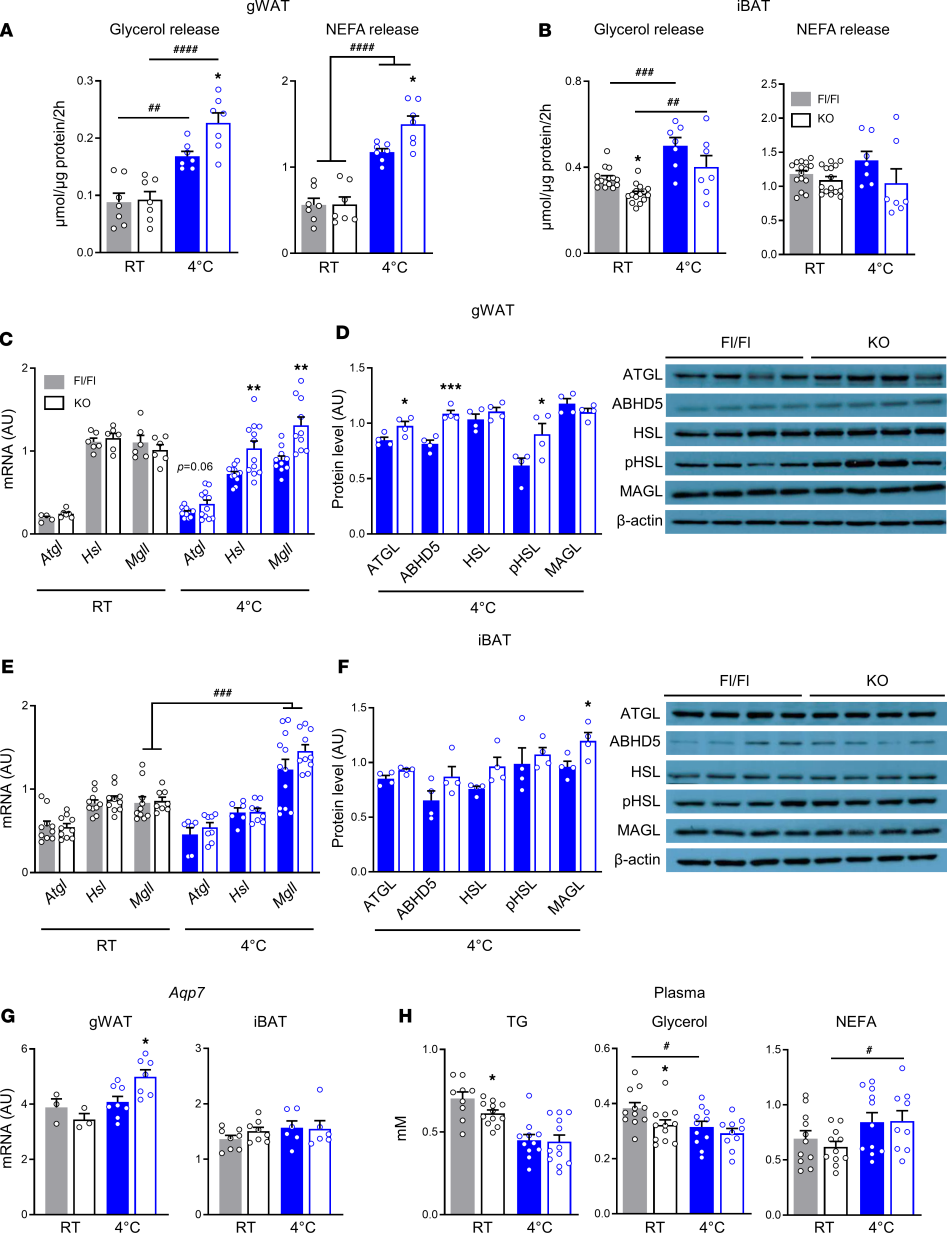
Increased cold-stimulated activity of the lipolytic arm of the GL/FFA cycle in gWAT of AT-ABHD6–KO mice. (**A** and **B**) Ex vivo glycerol and NEFA released from AT explants (7 mice/group). (**C**–**F**) Expressions of lipolytic enzymes at mRNA level (6–12 mice/group) and at protein level (4 mice/group). (**G**) Aquaporin 7 (*Agp7*) mRNA expression. (**H**) Plasma TG, glycerol, and NEFA levels (11–12 mice/group). Statistics were calculated by 1-way ANOVA and Tukey’s post hoc test, and for **C**–**F** to compare Fl/Fl vs. KO, Student’s *t* test was used. The effects of genotype: *P < 0.05, **P < 0.01, ***P < 0.001; the effects of cold induction: ^#^*P* < 0.05, ^##^*P* < 0.01, ^###^*P* < 0.001, ^####^*P* < 0.0001.

Twenty-four–hour cold stress induced ex vivo lipolysis in gWAT from both Fl/Fl and KO mice, measured as glycerol and FFA release. Cold-stimulated glycerol and FFA release was higher in ABHD6-ablated gWAT (*P* < 0.05), even though the basal levels at RT remained unchanged (Figure 6A). Thus, while acute (2 hours) ISO stimulation led to lowered lipolysis in ABHD6-deleted gonadal adipocytes versus control (Figure 1, C and F), 24 hours cold exposure enhanced the lipolysis in the KO gWAT (Figure 6A). Cold exposure of KO mice for 24 hours led to altered expression of GL/FFA cycle genes, as seen below, which could not have happened during acute ISO treatment ex vivo. Consistently, expression of lipolytic enzymes ATGL, HSL, and MAGL, at mRNA and protein levels, was elevated in gWAT from cold-induced AT-ABHD6–KO mice (Figure 6, C and D). Also, ABHD5 protein, an activator of ATGL, and activating phosphorylation of HSL (Ser563) were increased in the gWAT of KO mice (Figure 6D). Expression of aquaglyceroporin 7, the major glycerol transporter in adipocytes, was also increased at mRNA level in gWAT from cold-treated AT-ABHD6–KO mice (Figure 6G), probably to facilitate glycerol efflux during accelerated lipolysis. Lipolysis measured as glycerol and FFA release from explants of iBAT of the KO versus Fl/Fl mice was slightly reduced at RT and under cold (Figure 6B). Expression of most of the lipolytic enzymes in iBAT was unchanged between KO and Fl/Fl mice at RT and after cold (Figure 6, E and F). Notably, cold exposure led to elevated mRNA expression of MAGL (*Mgll*) in iBAT from both groups (Figure 6E). Also, it is interesting to note that ABHD6 mRNA and protein expression in control mice was markedly induced by cold in iBAT but not in gWAT (Supplemental Figure 5, A and B). Plasma analysis showed modest reductions in TG, glycerol, and NEFAs in KO mice at RT, with no apparent changes between the control and KO mice under cold (Figure 6H). Finally, as shown in Supplemental Figure 5C, gWAT and iBAT *18s* levels (used as a reference gene in this study) revealed stable mRNA levels across different experimental conditions (RT vs. cold).

### Adipose-specific deletion of ABHD6 does not alter β-adrenergic receptor 3–mediated lipolysis.

In order to further unravel the signaling pathways involved in enhanced expression and activation of lipases in the gWAT of cold-exposed AT-ABHD6–KO mice, we assessed the role of β-adrenergic receptors (β-ARs). β-AR3, encoded by *Adrb3*, is expressed predominantly in the fat tissues and is involved in WAT browning and BAT activation mostly through UCP1-dependent mechanisms (34). To investigate if stimulation of β-AR3–mediated lipolytic responses at RT can recapitulate the cold-induced changes in the lipolysis pathway, AT-ABHD6–KO and Fl/Fl mice were injected with a selective β-AR3 agonist (CL316243; 1 mg/kg/d). Plasma samples were collected at 0, 15, and 30 minutes of the first injection (acute response) and at the end of the protocol (2 days). Plasma glucose level decreased similarly in both groups by acute CL treatment (Supplemental Figure 5D). As expected, circulating glycerol, NEFA, and TG concentrations were all increased within 30 minutes postinjection in both groups, though a significantly lower level of TG was observed in the KO mice (Supplemental Figure 5, E–G). No significant differences were found in the ex vivo lipolysis either in gWAT or in iBAT (Supplemental Figure 5, H and I) between CL-treated AT-ABHD6–KO versus Fl/Fl mice. Consistently, plasma parameters (TG, glycerol, and NEFA) measured at the end of the protocol (2-day treatment) were comparable between groups (Supplemental Figure 5J). Under cold-stress condition, among the 3 β-ARs, only *Adrb2* mRNA level was significantly increased in the gWAT of AT-ABHD6–KO mice (Supplemental Figure 5K). Nevertheless, similar *Adrb3* mRNA levels between KO and Fl/Fl (Supplemental Figure 5, K and L) and comparable responses to the CL treatment in both genotypes suggest that β-AR3 signaling is likely not altered in the KO mice. However, whether the increase in gWAT *Adrb2* expression in cold-exposed ABHD6-KO mice is under the influence of the SNS, endocrine factors, or adipocyte autonomous signaling (MAG/PPAR-dependent) is not clear. Thus, we further investigated whether adipocyte β-AR expression is dependent on the activity of PPARs, because we found elevated *Ppara* and *Pgc1a* expression in gWAT and our earlier study indicated increased function of PPARs in whole-body ABHD6-KO mice (22). Differentiated 3T3-L1 adipocytes were treated with WY14643 (WY) and pioglitazone (Pio), agonists of PPARα and PPARγ, respectively, and the expression of β-ARs was assessed. Interestingly, only *Adrb2* expression was significantly induced in adipocytes treated with PPARα agonist (*P* < 0.001; Supplemental Figure 5M), which is in line with the cold exposure data. *Adrb3* mRNA expression was similar between DMSO and PPARα agonist–treated cells (Supplemental Figure 5M), whereas *Adrb1* mRNA was not detectable in 3T3-L1 adipocytes (data not shown). Taken together, the data suggest that enhanced cold tolerance with adipose ABHD6 deletion occurs independently of changes in β-AR3 agonism and that it might be conducted via a PPARα-β-AR2-GL/FFA cycle axis.

### Increased cold-stimulated activity of the lipogenesis arm of the GL/FFA cycle in gWAT of AT-ABHD6–KO mice.

**Figure 7:**
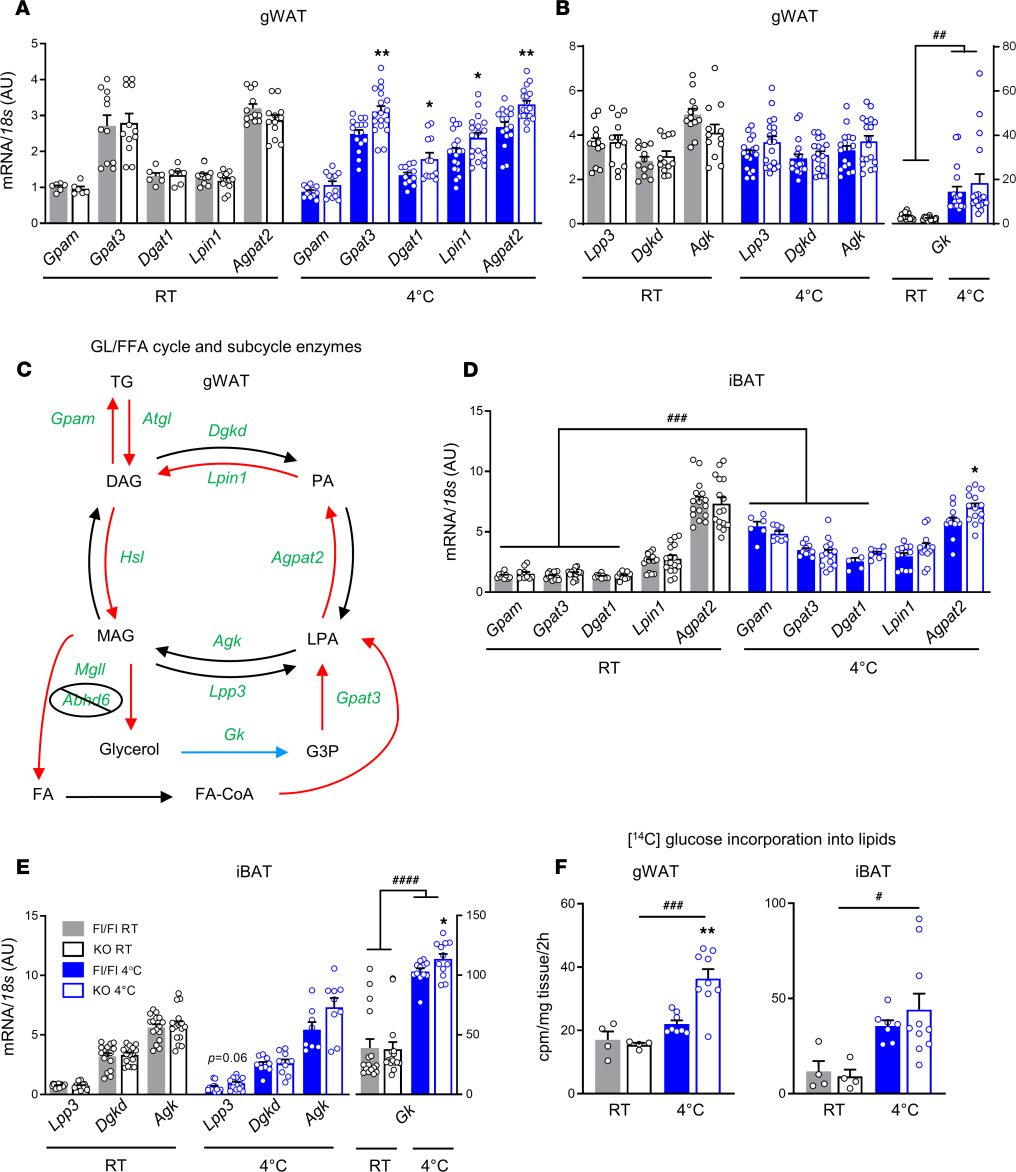
Increased cold-stimulated activity of the lipogenesis arm of the GL/FFA cycle in gWAT of AT-ABHD6–KO mice. (**A** and **B**) Gene expressions of lipogenic enzymes (6–18 mice/group). (**C**) GL/FFA cycle and subcycle enzyme gene expression levels in gWAT from cold-challenged mice. Red arrows correspond to the upregulated enzymes in the KO mice (vs. Fl/Fl), black arrows refer to enzymes with similar mRNA expressions between genotypes, and the blue arrow indicates the upregulation of the enzymes in response to cold, independent of genotype. (**D** and **E**) Gene expressions of GL/FFA subcycle enzymes (6–18 mice/group). (**F**) Ex vivo de novo lipogenesis (^14^C-glucose). Statistics by 1-way ANOVA and Tukey’s post hoc test, except (**A**–**D**) Student’s *t* test to compare Fl/Fl vs. KO. The effects of genotype: *P < 0.05, **P < 0.01; the effects of cold induction: ^#^*P* < 0.05, ^##^*P* < 0.01, ^###^*P* < 0.001, ^####^*P* < 0.0001.

As thermogenesis by GL/FFA cycling depends on both lipolysis and lipogenesis arms of this pathway, we examined if lipogenesis per se and expression of lipogenic genes is also altered in the KO mice under cold stress. Glycerol-3-phosphate acyltransferase 3 (*Gpat3*), diacylglycerol acyltransferase 1 (*Dgat1*), Lipin 1 (*Lpin1*), and lysophosphatidic acid acyltransferase (*Agpat2*) mRNAs were all upregulated in the gWAT from cold-exposed AT-ABHD6–KO mice (Figure 7A). Additional genes that contribute to GL/FFA subcycles, including lipid phosphate phosphatases (*Lpp3*), diacylglycerol kinase δ (*Dgkd*), and acylglycerol kinase (*Agk*), were similarly expressed in both Fl/Fl and KO mice at RT and after cold stress (Figure 7B). In accordance with previous reports (35), glycerol kinase (Gk) expression in gWAT was induced in response to cold, without difference between the 2 groups of mice (Figure 7B). Figure 7C summarizes all the observed changes in the expression of GL/FFA cycle and subcycle enzyme genes in gWAT of cold-challenged mice. The expression of the lipogenic enzymes glycerol-3-phosphate acyltransferase, mitochondrial (*Gpam*); *Gpat3*; and *Dgat1* and subcycle enzyme *Gk* in iBAT were increased in both KO and Fl/Fl mice after cold exposure (Figure 7, D and E). Expression of *Gk* and *Agpat2* was slightly but significantly increased in the KO versus Fl/Fl iBAT after cold exposure (Figure 7, D and E).

Notably, in iBAT from both genotypes, cold exposure led to elevated mRNA expression of *Mgll*, some lipogenic enzymes, and *Gk*, which suggests increased intracellular reusage of lipolytic glycerol in iBAT. In agreement with the gene expression changes, ex vivo de novo lipogenesis from glucose was significantly higher in the gWAT from the cold-exposed KO mice versus Fl/Fl (*P* < 0.01; Figure 7F). Although lipogenesis in BAT explants was induced by cold, no difference was found between genotypes, regardless of temperature (Figure 7F). Moreover, there were no changes between groups following 2-day treatment at RT with β-AR3 agonist CL in body weight, rectal temperature, blood glucose, or gWAT and iBAT de novo lipogenesis (Supplemental Figure 6, A–D).

### 2-MAG as the molecular signal for the cold-tolerance phenotype of AT-ABHD6–KO mice.

**Figure 8:**
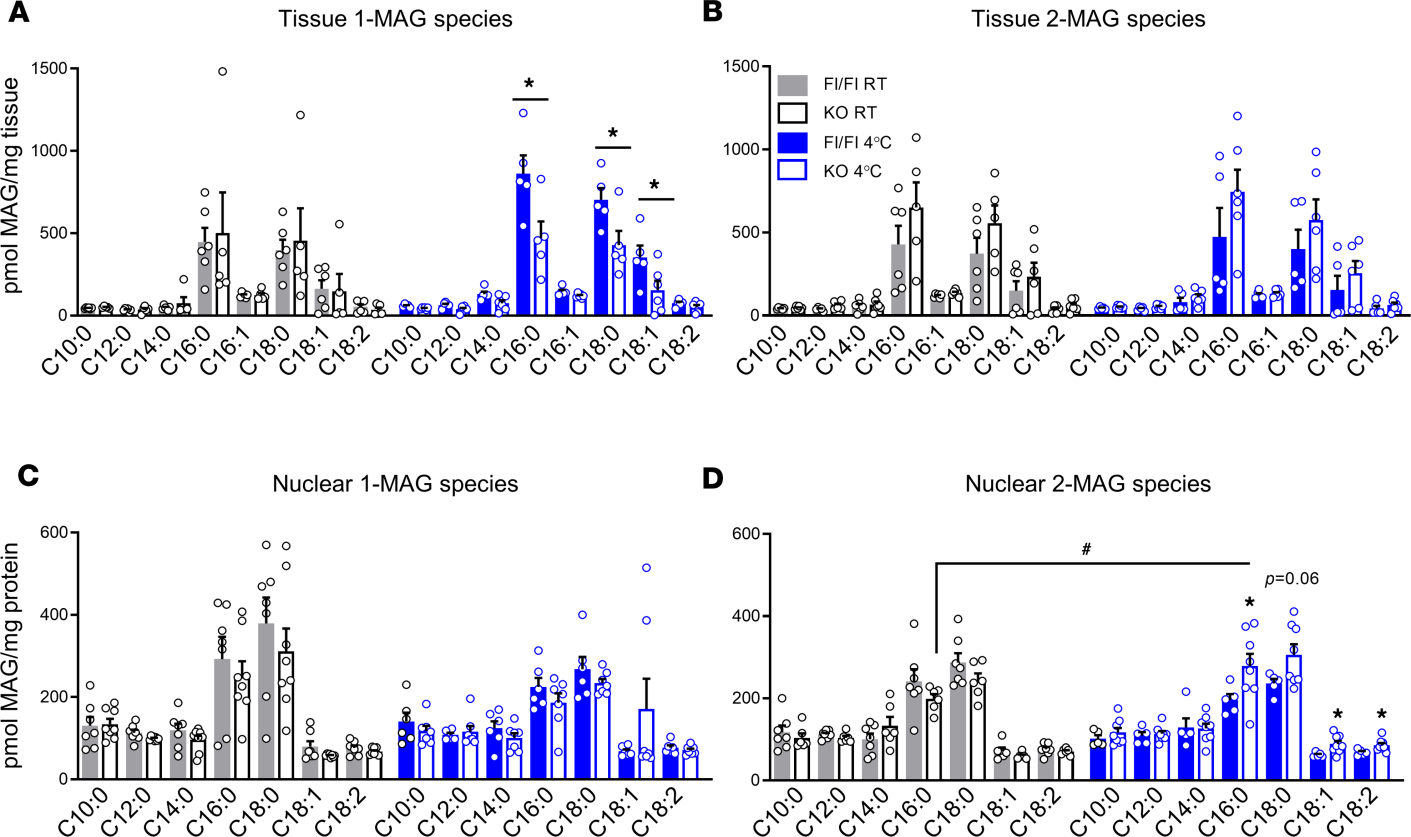
Nuclear 2-MAG levels increase in visceral WAT of cold-challenged KO mice. (**A** and **B**) Analysis of 1-MAG and 2-MAG species in the whole tissue of gWAT. (**C** and **D**) Analysis of 1-MAG and 2-MAG species in the isolated nuclear fraction from gWAT. *n* = 5–8 mice/group. Statistics by 1-way ANOVA and Tukey’s post hoc test. The effects of genotype: *P < 0.05; the effects of cold induction: ^#^*P* < 0.05.

We further examined the mechanism by which adipose ABHD6 deletion leads to greater EE in response to cold, considering that the thermogenic/lipid metabolism regulatory genes, PPARα/γ and PGC1α, were upregulated only in the gWAT and not iWAT and BAT of cold-exposed KO mice. In order to assess whether MAG-mediated signaling is involved, we first analyzed whole-tissue 1-MAG and 2-MAG species in gWAT of KO and Fl/Fl mice at both temperature conditions. As shown in Figure 8A, levels of 1-MAG species at RT were similar between the 2 genotypes, but a slight reduction in 1-MAG species (C16:0, C18:0, and C18:1) was observed upon cold exposure in the AT-ABHD6–KO versus Fl/Fl mice. On the other hand, levels of all 2-MAG species (particularly C16:0, C18:0, and C18:1) were found to be marginally elevated in ABHD6-deficient gWAT at both temperatures (Figure 8B).

**Figure 9:**
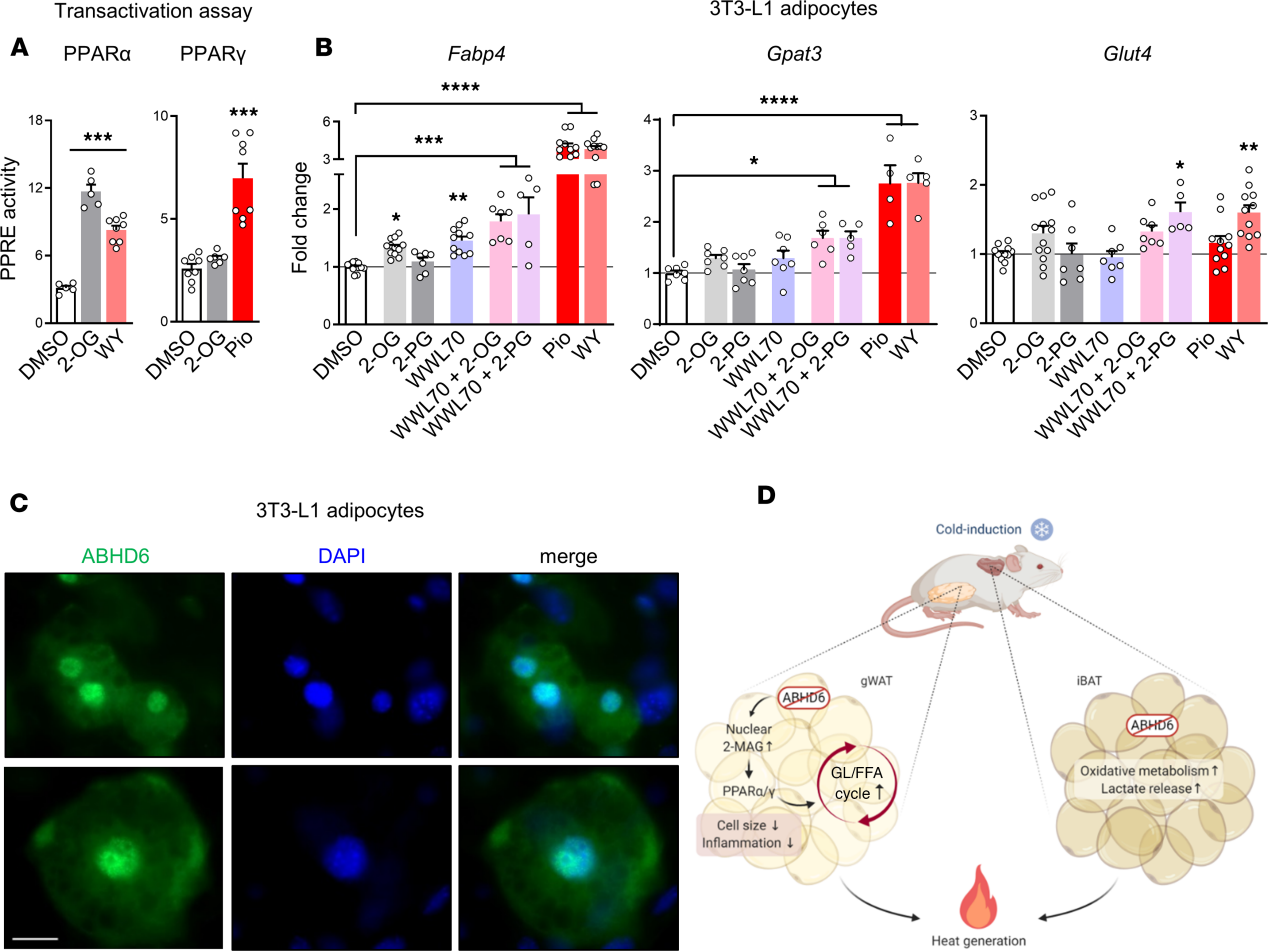
2-MAG as the molecular signal for the cold tolerance phenotype of AT-ABHD6–KO mice. (**A**) Transactivation of PPARα and PPARγ by 2-oleoylglycerol (2-OG) in HEK293T cells, as well as WY-14643 (WY) and pioglitazone (Pio). PPAR response element–driven expression of luciferase was measured following the activation of test compounds to the cells. Data are expressed as relative luciferase activity. (**B**) Expression of PPARγ target genes was measured 24 hours after treatment of 3T3-L1 adipocytes with 2-OG, 2-palmitoylglycerol (2-PG), and WWL70, as well as WY and Pio. (**C**) Representative immunofluorescence staining for ABHD6 localization in differentiated 3T3-L1 adipocytes. The cells are stained with anti-ABHD6 antibody (green), and DAPI is used for nuclei staining (blue). Images were acquired at 60× and scale bar is 60 μm. (**D**) Schematic overview of sympathetic activation of brown and white adipocytes, in the absence of ABHD6. Cold stimulation of AT-ABHD6–KO mice leads to the accumulation of the signaling pool of nuclear 2-MAG in the gWAT, resulting in the activation of PPARs, which causes the upregulation of adipose antiinflammatory markers and lipogenesis and lipolysis genes and thus thermogenic GL/FFA cycling. ABHD6-deleted brown adipocytes showed enhanced oxidative and glucose metabolism. Two independent experiments; *n* = 5–12. All statistics by 1-way ANOVA and Tukey’s post hoc test (*P < 0.05, **P < 0.01, ***P < 0.001, ****P < 0.0001).

ABHD6 and MAGL have been postulated to modulate distinct subcellular pools of MAG (18, 20). As described above, we found elevated levels of *Ppara/g* nuclear receptors and their coactivator, PGC1α, in association with accelerated GL/FFA cycle in gWAT from cold-induced KO versus Fl/Fl. Interestingly, previous studies documented the existence of active lipid metabolism in the nucleus, where the lipid mediators/messengers efficiently regulate various signaling processes (36–38). Therefore, we examined nuclear 1- and 2-MAG species levels and whether MAG can activate PPARα and -γ in the gWAT from cold-exposed KO mice. For this, we isolated gWAT nuclei and ascertained their purity based on lamin A/C enrichment (Supplemental Figure 7A). MAG species analysis in the nuclear fraction revealed similar 1-MAG species levels between control and KO mice at both RT and 4°C (Figure 8C). However, as shown in Figure 8D, 2-MAG species (C16:0, C18:0, C18:1, and C18:2) were significantly elevated in the nuclear fraction from gWAT of cold-exposed KO mice. This indicates that ABHD6 deletion in gonadal adipocytes leads to a rise in a distinct pool of long chain fatty acyl groups containing 2-MAG species localized in the nucleus, where it can act as a ligand to PPARs and activate them. Although ABHD6 association with the nuclear membrane has been previously proposed (39, 40), here we provide evidence for its nuclear localization by fluorescence microscopy in differentiated 3T3-L1 adipocytes (Figure 9C). Thus, it is possible that the nuclear pool of MAG is regulated by ABHD6 localized in nuclei.

We next examined if 2-MAG can activate PPARs, similar to 1-MAG (22). We tested the effect of 2-oleoylglycerol (2-OG) in PPAR transactivation assay in HEK293T cells. Similar to PPARα agonist WY, 2-OG markedly enhanced the PPARα-driven luciferase gene expression (*P* < 0.001), and such activation was not seen in PPARγ transactivation assay (Figure 9A). Thus, 2-MAG can directly activate PPARα but not PPARγ. Hepatocytes harbor significant levels of PPARα, while PPARγ is predominantly expressed in adipocytes. Therefore, mouse hepatocytes and 3T3-L1 adipocytes were used for investigating 2-MAG/PPAR activation by following expression of target genes of PPARs. Treatment of hepatocytes with exogenous 2-OG resulted in a marked increase of PPARα/γ target genes’ mRNAs, including fatty acid binding protein 4 (*Fabp4*, PPARγ target, refs. 41, 42), carnitine palmitoyltransferase 1A (*Cpt1a*, PPARα target, ref. 43), and *Gk* (PPARα and -γ target, ref. 44), comparable with the effects of PPARα and PPARγ agonists (WY and Pio, respectively, and Supplemental Figure 7B). Because ABHD6 is highly expressed in the differentiated fat cells, and because adipocytes have high lipid content, an ABHD6 inhibitor, WWL70, was added along with 2-MAGs to facilitate their delivery and accessibility to the nuclear receptors, without being hydrolyzed. Therefore, we treated 3T3-L1 adipocytes with 2-OG, 2-palmitoylglycerol (2-PG), WWL70, and PPAR agonists and evaluated the expression profiles of the 3 known PPARγ target genes in adipocytes, *Fabp4* (45), *Gpat3* (46), and *Glut4* (47). All 3 genes were clearly induced by the combination of WWL70 with 2-OG or 2-PG (Figure 9B). Thus, 2-MAG is capable of directly activating PPARα and inducing the expression of PPARα target genes and also PPARγ target genes, probably indirectly.

## Discussion

UCP1-deficient mice are severely sensitive to acute cold challenge;however, they can survive when gradually acclimated to cold temperatures (6), which suggests the presence of UCP1-independent thermogenic mechanisms. Several alternative thermogenic pathways in white and brown adipocytes have been proposed, including creatine (8) and calcium (6) futile cycles, and glycerol-3-phosphate shuttle and lipid turnover (5, 10). In the present study we provide evidence that adipose-specific deletion of ABHD6 in adult mice does not induce major phenotypic changes either at RT or under thermoneutral conditions but (a) confers resistance to cold-induced hypothermia, at least in part by stimulating thermogenic GL/FFA cycle in visceral fat as demonstrated by gene/protein expression measurements and functional assays, in particular the simultaneous increased lipogenesis and lipolysis pathways and the induction of genes/enzymes of both the lipogenesis and lipolysis arms of the GL/FFA cycle; (b) increases 2-MAG levels in gWAT, which contribute to enhanced MAG/PPAR signaling and to elevated expression of GL/FFA cycle enzymes; (c) does not alter cold-induced adipose UCP1 expression and β-AR3–mediated metabolic responses compared with control mice, despite inducing cold tolerance; and (d) enhances glucose and oxidative metabolism in BAT, without changes in the GL/FFA cycle gene/protein expressions and lipid turnover in this tissue.

Continuous adrenergic stimulation has been suggested to recruit both lipolysis and lipogenesis pathways to sustain cellular energy production (32, 48) but with the resultant decline or stabilization in lipid stores. We found a similar situation in visceral fat in the AT-ABHD6–KO mice following 24 hours cold exposure, where the lipolysis and lipogenesis arms of the GL/FFA cycle were enhanced simultaneously, causing extensive remodeling of gonadal adipocytes into smaller and metabolically more active cells. The elevated cold-stimulated lipolysis in the gWAT from AT-ABHD6–KO mice was associated with enhanced expression/activity of lipolytic enzymes (ATGL, ABHD5, HSL/phosphorylated HSL, MAGL). In addition, the expression of lipogenic enzymes (*Gpat3*, *Dgat1*, *Lpin1*, and *Agpat2*) was also found to be elevated in the gWAT of cold-exposed KO mice. Furthermore, the current data revealed enhanced expression of PPARα, PPARγ, and PGC1α in the gWAT (but not in iWAT or iBAT) of cold-challenged AT-ABHD6–KO versus control mice. Thus, the increased expression of GL/FFA cycle enzymes is likely mediated through PPARs/PGC1α, which are known to regulate lipid and energy metabolism (49, 50), and indeed we found induction of several PPAR target genes only in the visceral fat depot of the cold-induced KO mice.

Contribution of iBAT to the enhanced EE in the cold-challenged KO mice appears to be limited to increased glucose uptake and oxidative metabolism, in vivo, as revealed by PET/CT analysis in AT-ABHD6–KO versus Fl/Fl mice, which was also confirmed ex vivo, by the elevated glucose oxidation, glucose utilization, and glycolysis. Considering that the absence of ABHD6 had no marked impact on de novo lipogenesis in iBAT, it seems that most of the internalized glucose was utilized for energy generation processes. Also, the enhanced expression of *Mgll* and *Gk* in iBAT of cold-challenged KO mice, without any concomitant changes in lipogenic enzymes, likely diverts lipolysis-derived glycerol toward glycolytic conversion to lactate, causing a decline in lipid stores. Nevertheless, the precise mechanism underlying this phenotype is uncertain.

PPARα has been suggested to play a role in the regulation of membrane trafficking of β-AR2 and β-AR3, which control adipose lipolysis (51). Thus, upon PPARα activation in 3T3-L1 adipocytes, we noticed a significant increase of *Adrb2* expression (*P* < 0.001). Also, the higher mRNA expression of *Adrb2*, but not *Adrb1* or *Adrb3*, in the gWAT of cold-challenged KO versus Fl/Fl mice suggested the possible activation of β-AR2–mediated signaling and lipolysis in the AT-ABHD6–KO mice. Although β-AR3 is known as the most abundant adrenergic receptor in rodent adipose tissue, β-AR2 has also been reported to regulate cold-stimulated energy expenditure (52). The administration of β-AR3 agonist (CL316243) in vivo resulted in similar responses between the control and AT-ABHD6–KO mice at RT, suggesting that β-AR3 may not have a major role in cold-induced thermogenesis in the mice deficient in ABHD6 in adipose tissues. Thus, it is plausible that the increased energy expenditure of the AT-ABHD6–KO mice under cold stress is partly due to enhanced β-AR2 signaling in gWAT, mediated via PPARα activation.

Considering that MAG activates PPARα/γ (22), that activation of transcription factors is relevant in the nucleus, and that adipocyte ABHD6 is localized in nucleus, we measured MAG levels in isolated nuclei from gWAT. We found elevated 2-MAG species, in particular, C16:0, C18:0, and to a lesser extent C18:1 and C18:2, in the cold-exposed KO mice. In fact, 2-MAG was found to be capable of transactivating PPARα (Figure 9A), as well as inducing PPARα and PPARγ target genes in adipocytes, in vitro (Figure 9B). However, we did not see a direct transactivation of PPARγ by 2-OG. Given the central role of PPARγ in adipocyte metabolism (53), and because many enzymes of the lipogenic pathway, which are under the direct control of PPARγ, are induced in the gWAT of cold-exposed KO mice, it is possible that different 2-MAG species (other than 2-OG) or distinct lipids or mechanisms are responsible for PPARγ activation. Supporting this view, a recent study reported that PPARα/γ shared target genes are relevant for brown adipocyte cold-induced thermogenesis (54). Finally, the model depicted in Figure 9D summarizes the present results, showing that adipocyte-specific deletion of ABHD6 leads to accelerated GL/FFA cycle in the gWAT via 2-MAG/PPARα activation and contributes to cold-induced thermogenesis, as 7 ATP molecules are hydrolyzed for each turn of this cycle (7). Thus, our findings implicate an important, but less explored, thermogenic role of gWAT in response to the cold stress.

In this study, using the *Adipoq-Cre*/ERT2 transgene, ABHD6 was specifically deleted in all mature adipocytes within both white and brown depots. Thus, it is not possible to decipher a depot-specific role of ABHD6 on whole-body measurements, such as glucose and insulin tolerance or energy expenditure. To overcome this limitation, ex vivo experiments were done separately on BAT and WAT explants, and this allowed us to identify the thermogenic role of ABHD6 in both gWAT and iBAT. It will be of interest in the future to perform cold-induced thermogenesis studies using adipose depot-specific ABHD6-KO mouse models in order to better delineate and quantify in vivo the respective roles of gWAT and iBAT ABHD6 in the regulation of thermogenesis.

In conclusion, we identified a previously unrecognized cold-induced thermogenesis program in the gWAT that involves 2-MAG/PPARα signaling and accelerated thermogenic GL/FFA cycle. The contribution of this thermogenic mechanism was revealed by the suppression of adipose ABHD6, which negatively regulates 2-MAG levels. Like any other physiological function, cold-induced thermogenesis is tightly regulated with both positive and negative modulators (55). Our data demonstrate that adipocyte ABHD6 acts as a brake for thermogenesis in the visceral fat to fine-tune cold-induced heat production and to prevent the loss of adipocyte lipid stores. Cold-induced thermogenesis has therapeutic potential for treating diet-induced obesity, insulin resistance, and dyslipidemia (56, 57). Hence, pharmacological suppression of adipocyte ABHD6 could open new therapeutic avenues to treat obesity-associated metabolic diseases by enhancing energy expenditure.

## Methods

### Animals

Mice were housed at RT (22°C) on a 12-hour light/12-hour dark cycle with ad libitum access to water and food. *Abhd6*^fl/fl^ homozygous mice on a C57BL/6N background were generated as described before (20). Homozygous floxed mice were bred with *Adipoq-Cre*/ERT2 mice, on C57BL/6N background (25), to generate heterozygous *Abhd6*^fl/+^ *Adipoq-Cre*/ERT2, which were bred a second round to generate WT, homozygous *Abhd6*^fl/fl^ *Adipoq-Cre* (to obtain AT-ABHD6–KO mice by TMX injection), homozygous *Abhd6*^fl/fl^ (Fl/Fl), and *Adipoq-Cre* (Cre) mice. At 8 weeks of age, Fl/Fl, Cre and AT-ABHD6–KO (KO) male mice were given TMX (80 mg/kg body weight, dissolved in 10% ethanol in corn oil; oral gavage) every other day, for 3 cycles. After TMX treatment, mice were fed a normal diet for 12 weeks, and body weight and food intake were measured weekly.

### Cold exposure

KO, Cre, and Fl/Fl male mice at the age of 14–16 weeks were randomly divided into 2 groups, one kept at RT and the other in the cold. The cold group mice were kept at 4°C for either 3 hours or 24 hours. For acute cold exposure (3 hours), mice were singly housed in prechilled cages without food and bedding but with free access to water. During 24 hours cold induction, mice were individually housed with minimum bedding on a 12-hour light/12-hour dark cycle with ad libitum access to chow diet and water. Blood samples were collected and rectal temperature was recorded.

### β-AR3 stimulation by CL316243 treatment

KO and Fl/Fl male mice (14–16 weeks old) were treated with CL316243 (MilliporeSigma; 1 mg/kg/d) by intraperitoneal injection for 2 days. Blood samples were collected and rectal temperature was recorded before and after treatment. For acute treatment, mice were fasted overnight, and blood was collected from the tail vein before and at 15 and 30 minutes after CL316243 injection.

### In vivo metabolic studies

#### Glucose tolerance test (OGTT).

OGTT was conducted in 17-week-old KO, Cre, and Fl/Fl mice. After 6 hours of food withdrawal, glucose (2 g/kg body weight) was administered orally. Blood was collected from the tail vein for glucose and insulin monitoring before gavage and at 15, 30, 60, 90, and 120 minutes after gavage.

#### Insulin tolerance test (ITT).

ITT was performed in 20-week-old KO, Cre, and Fl/Fl mice. After 4 hours of food withdrawal, insulin (0.75 U/kg body weight, Humulin; Lilly) was administered intraperitoneally, and blood was collected from the tail vein for glucose monitoring prior to and at 15, 30, 45, 60, 90, and 120 minutes after injection.

#### Comprehensive Lab Animal Monitoring System (CLAMS).

Whole-body energy metabolism was assessed by individually placing KO, Cre, and Fl/Fl mice (16 weeks old; chow diet) in metabolic chambers (CLAMS, Columbus Instruments) for 3 days (24 hours of adaptation at 22°C, 24 hours at 22°C, and 24 hours at 4°C) or for 5 days at 30°C for studying the effect of thermoneutral conditions. Two weeks prior to the experiment, mice were surgically implanted with intraperitoneal temperature probes (G2 HR E-mitter, Bio-Lynx) to continuously monitor core body temperature. After complete recovery from surgery, the mice were placed in CLAMS. Core body temperature and other parameters, including oxygen consumption (VO_2_), carbon dioxide production (VCO_2_), food/water intake, and physical activity, were monitored at regular intervals. EE was expressed as a function of metabolic mass (lean mass + 0.2 fat mass) (58). Body composition (lean and fat mass) was measured by magnetic resonance (EchoMRI Analyzer-700).

#### Imaging (PET/CT).

Male AT-ABHD6–KO and Fl/Fl mice underwent a randomized (1:1) crossover study at 30°C (isothermic) versus 10°C (hypothermic) for 48 hours prior to sequential μPET dynamic imaging with [^11^C]-acetate and [^18^F]-FDG after overnight fasting, with 1-week washout period at normal housing conditions at RT, in between the 2 experimental conditions. μPET experiments were performed under anesthesia (isoflurane 2.0%, 1.5 L/min), delivered to the animal through a nose cone. In the hypothermic condition, mice were injected with the β-AR3 agonist CL316243 (2 mg/kg) prior to the PET tracer injection to maintain cold-induced BAT stimulation (16). All the PET tracers were injected through the tail vein, and the imaging was performed with the avalanche photodiode-based small-animal μPET scanner (LabPET, Triumph, Gamma Medica) at the Sherbrooke Molecular Imaging Center (Centre de recherche du CHUS, Université de Sherbrooke). Anesthetized mice were placed on the scanner bed and positioned with the heart centered within the field of view of the scanner. A bolus of [^11^C]-acetate (10 MBq, in 0.2 mL of 0.9% NaCl) was injected intravenously followed by a 20-minute dynamic μPET data acquisition. Then, a bolus of [^18^F]-FDG (10 MBq, in 0.1 mL of 0.9% NaCl) was injected, and a 30-minute PET acquisition was done. Residual [^11^C]-acetate activity during [^18^F]-FDG acquisition was corrected by acquiring a 60-second frame prior to the injection of [^18^F]-FDG, accounting for the disintegration rate of [^11^C]. Low-dose CT scan imaging was performed using the integrated X-O small-animal CT scanner of the Triumph, Gamma Medica, platform, consisting of a 40 W x-ray tube with a 75 μm focal spot diameter and a 2240 × 2368 CsI flat panel x-ray detector. All the obtained images were analyzed as described previously (16). Tissue oxidative metabolism index (the rapid fractional tissue clearance of [^11^C]-acetate) was determined from tissue [^11^C] activity over time using monoexponential fit from the time of peak tissue activity. Tissue glucose fractional extraction (Ki, i.e., the fraction of circulating glucose taken up by the tissue over time) was determined using the Patlak graphical analysis of [^18^F]-FDG activity. Tissue glucose uptake (Km) was determined by multiplying Ki by the plasma glucose concentration (16).

### Mouse adipose depots

For further analysis, gWAT, iWAT, and iBAT fat depots were dissected (Supplemental Figure 1D).

### Cell culture and treatment

#### 3T3-L1 preadipocyte differentiation.

3T3-L1 preadipocytes (American Type Culture Collection, ATCC) were differentiated according to standard procedures as previously described (59).

#### Primary culture of hepatocytes.

Primary hepatocytes were isolated from male WT mice first by in situ perfusion with a washing solution, then by pumping a collagenase solution (MilliporeSigma C5138) to digest the liver. After the isolation of viable cells, hepatocytes were seeded in DMEM with 25 mM glucose, supplemented with 10% FBS and antibiotics.

#### Transactivation assays to assess PPAR promoter activation.

HEK293T cells (ATCC) were maintained in DMEM supplemented with 10% (*v/v*) FBS and antibiotics. At approximately 60% confluence, the HEK293T cells were transfected with the plasmids expressing PPARα or PPARγ and PPAR response element-directed luciferase expression plasmid (PPRE-X3-TK-luc) and Renilla luciferase control plasmid. Transactivation assay was performed as previously explained (22).

### Ex vivo metabolic studies

All the ex vivo metabolic studies were done on 14- to 16-week-old male Fl/Fl and KO mice.

#### Lipolysis.

Lipolysis was assessed by glycerol and NEFA release from collagenase-isolated mature adipocytes or tissue explants, under basal and stimulated (ISO, MilliporeSigma, 1 μM) and CL316243 (MilliporeSigma, 10 μM) conditions, as previously described (60). Commercial kits were used to measure glycerol (MilliporeSigma) and NEFA release (Wako NEFA-HR, FUJIFILM).

#### De novo lipogenesis.

Adipose tissue explants were incubated in in-house Krebs-Ringer bicarbonate HEPES buffer (KRBH), supplemented with 2% fatty acid–free BSA, 4 mM glucose, and 0.4 μCi/mL d-[U-^14^C] glucose (250 mCi/mmol) at 37°C in a humidified incubator (5% CO_2_) for 2 hours. Glucose incorporation into lipids was then determined by total neutral lipid extraction with Folch method, followed by radioactivity assessment. Results were normalized to the weight of the tissue.

#### Glucose oxidation and utilization.

Freshly isolated tissue explants were washed, minced, and transferred to capless tubes (T1) in KRBH, 2.5% fatty acid–free BSA containing 0.5 μCi of d-[5-^3^H] glucose (16 Ci/mmol), and 1 μCi/mL D-[U-^14^C] glucose (250 mCi/mmol) and 5 mM glucose. T1 tubes were transferred to the scintillation vials containing 1 M HCl and incubated at 37°C for 90 minutes. At the end of incubation, another capless tube (T2) containing 5% KOH was placed next to the T1, and the termination buffer (400 mM citrate/NaOH, pH 4.9, supplemented with antimycin A 10 mM, rotenone 10 mM, and potassium cyanide 5 mM) was added directly to the T1 tubes. After 1 hour of further incubation at RT, T2 tubes were removed, and the trapped ^14^CO_2_ in the KOH was measured by liquid scintillation counting to assess glucose oxidation (61). Following a 40-hour equilibration at RT, T1 tubes were also removed (stored for protein content), and the amount of ^3^H_2_O in HCl in the scintillation vials was assessed as an indicator of glucose usage (61). Results were normalized to the protein content.

#### Glucose uptake.

Adipose tissues were isolated, rinsed, weighed and basal and insulin-stimulated glucose uptake was measured using radioactive deoxyglucose (62).

#### Lactate release and tissue content.

To assess lactate release, explants of iBAT were incubated in 1 mL of KRBH, supplemented with 2.5% fatty acid–free BSA, pH 7.4, at 37°C for 2 hours in the presence of 5 mM glucose. At the end of the incubation, media were collected and frozen for lactate assay. To measure lactate tissue content, freshly isolated iBAT tissue samples were weighed and homogenized in about 20 volumes of chilled acetone/water mixture. The homogenate was centrifuged (30 minutes, 4°C, 1440*g*), and the precipitate and the floating lipid layer were discarded. The acetone extract (tissue lactate content) and media (released lactate) were used for lactate quantification (Cayman Chemical), and the data are expressed as mmol/g of tissue.

#### Extracellular acidification rate.

ECAR of BAT explants was measured using an XF24 analyzer (Seahorse Bioscience). BAT was collected and cut into approximately 10-mg-size pieces. BAT explants were washed with Seahorse assay buffer supplemented with 25 mM glucose, 2 mM glutamine, and 1 mM sodium pyruvate, adjusted to pH 7.4. One piece of tissue was placed in each well of a Seahorse XF24 islet capture microplate containing 525 μL of Seahorse assay buffer. After a 30-minute incubation at 37°C without CO_2_, ECAR was measured and normalized by protein content (BCA protein assay kit, Thermo Fisher Scientific).

### Mitochondrial preparations and oxygen consumption

iBAT mitochondria were prepared from freshly dissected tissue (63), and the protein content was measured. Mitochondria were suspended in an assay medium (MAS: 70 mM sucrose, 220 mM mannitol, 5 mM KH_2_PO_4_, 5 mM MgCl_2_, 2 mM HEPES, 1 mM EGTA, 0.2% fatty acid–free BSA, pH 7.2), and 10 μg of mitochondrial protein (in 50 μL) was loaded per well in Seahorse XF24 assay plates. To promote adherence, the plate was centrifuged at 4°C, 20 minutes, at 2000*g*, and 475 μL of MAS was carefully delivered to each well without disturbing the settled mitochondria. The plate was placed at 37°C for 4 minutes to equilibrate the temperature and then transferred to the XF24 Analyzer (Seahorse Bioscience). To measure the OCR, the following substances were injected into the wells, sequentially (final concentrations): 5 mM pyruvate and 5 mM malate, 3.5 mM ADP, 4 μM FCCP, and 5 μM each antimycin A and rotenone.

### Analysis of MAG species

Analysis of 1- and 2-MAG species was done as described previously (20). In brief, total lipids from adipose samples were extracted by Folch method. 1-MAG and 2-MAG were separated on thin-layer chromatography plates, and the corresponding bands were scraped and processed for FFA species analysis by HPLC. Individual quantities of MAG species were assessed from the measured FFA.

### Nuclear isolation and MAG analysis

Nuclei were purified from freshly dissected gWAT (64), with slight modifications. Minced adipose tissue was added to 4 volumes (*w/v*) of 0.3 M sucrose buffer (0.3 M sucrose, 20 mM KH_2_PO_4_, 20 mM Na_2_HPO_4_ at pH 7.2, 137 mM NaCl, 3 mM KCl, 0.02% Triton X-100), plus protease and phosphatase inhibitors (1 mM phenylmethylsulfonyl fluoride, 2 μg/mL aprotinin, 0.7 μg/mL pepstatin, 0.5 μg/mL leupeptin, and 10 mM sodium orthovanadate), and homogenized in an ice-cold Dounce homogenizer. The homogenate was filtered through a 70 μm cell strainer (Corning Life Sciences) and then centrifuged through a 1.4 M sucrose cushion. The nuclear pellet underneath the sucrose layer was gently resuspended in about the same volume of 0.3 M sucrose buffer. The purity of isolated nuclear fraction was determined using immunoblotting for specific organelle markers, such as lamin A/C (nuclear), GAPDH (cytosolic), and CII/CIII (mitochondrial). Isolated nuclei were used for lipid extraction by Folch method, followed by separation of MAG species and HPLC quantification, as detailed above.

### Tissue histology and analysis

Freshly dissected adipose tissues were fixed (10% paraformaldehyde), dehydrated, embedded in paraffin, and sectioned. Sections were stained with H&E and examined for UCP1 expression (anti-UCP1 antibody from Abcam). Images were processed for cell size and number using ImageJ Version 1.48v (National Institutes of Health, Bethesda, Maryland, USA). Utilizing a semiautomated method (65), the average area, frequency distribution, and number of adipocytes in the gWAT were estimated. iBAT lipid droplet size was also assessed. Briefly, images were converted to 8-bit gray scale with the threshold adjusted to mid gray scale range, converted to binary, and segmented with watershed plug-in; and finally, the lipid droplet particles were analyzed automatically.

### Immunofluorescence microscopy

3T3-L1 adipocytes were serum-starved (3 hours), fixed (4% paraformaldehyde), permeabilized, (100 μM digitonin), and incubated with anti-ABHD6 antibody (Cell Signaling Technology) and Alexa Fluor 488–conjugated secondary antibody (Thermo Fisher Scientific) (antibodies are all listed in Supplemental Table 1). Pictures were taken using fluorescence microscope and analyzed by ImageJ Version 1.48v (National Institutes of Health, Bethesda, Maryland, USA).

### Immunoblotting

Tissues were digested with lysis buffer (20 mM Tris-HCl, pH 7.2, containing 150 mM NaCl, 1 mM EDTA, 1 mM EGTA, 1% *v/v* Triton X-100, 0.1% SDS, and protease inhibitors), and after protein quantification, 25–40 μg protein was used for Western blot analysis. Antibodies are listed in Supplemental Table 1.

### RNA extraction and quantitative PCR

Total RNA was isolated from tissues/cells using the RNeasy Mini Kit (Qiagen). Following reverse transcription of 2 μg RNA to cDNA, gene expression was determined by quantitative PCR using SYBR Green. All gene expression analyses were run in duplicate and normalized to *18s*. Primer sequences are listed in Supplemental Table 2.

### Plasma analysis

Glycerol, NEFAs, and triglycerides were measured using commercially available kits (Supplemental Table 1).

### Statistics

Results are expressed as means ± SEM. All data were analyzed and figures were prepared using GraphPad Prism 6 (GraphPad Software). Statistical differences between 2 groups were assessed by unpaired, 2-tailed Student’s *t* test and between multiple groups using 1-way or 2-way ANOVAs, as indicated. A *P* value of less than 0.05 was considered statistically significant.

### Study approval

All the procedures for mice studies were performed in accordance with the Institutional Committee for the Protection of Animals at the University of Montréal CRCHUM. PET/CT-related experiments were approved by the Ethical Committee for Animal Care and Experimentation of the University of Sherbrooke.

## Author contributions

PP, SRMM, and MP designed research; PP, CA, YM, AAM, AG, CS, SZ, JG, RL, HE, IC, and CN performed research; PP, MLP, EJ, ACC, CN, SRMM, and MP analyzed data; and PP, SRMM, and MP wrote the paper.

## Supplementary Material

Supplemental data

## Version 1 11/17/2020

In-Press Preview

## Version 2 12/17/2020

Electronic publication
