# Supplementary material for: Adipose ABHD6 regulates tolerance to cold and thermogenic programs
Source: JCI Insight. 2020 Dec 17;5(24):e140294. doi: 10.1172/jci.insight.140294 (PMC7819748; doi:10.1172/jci.insight.140294)
Supplement: Supplemental data [file jciinsight-5-140294-s042.pdf]

SUPPLEMENTAL INFORMATION

Adipose ABHD6 regulates tolerance to cold and thermogenic programs

Pegah Poursharifi, Camille Attané, Yves Mugabo, Anfal Al-Mass, Anindya Ghosh, Clémence Schmitt, Shangang Zhao, Julian Guida, Roxane Lussier, Heidi Erb, Isabelle Chénier, Marie-Line Peyot, Erik Joly, Christophe Noll, André C. Carpentier, S.R. Murthy Madiraju, Marc Prentki

SUPPLEMENTAL FIGURES

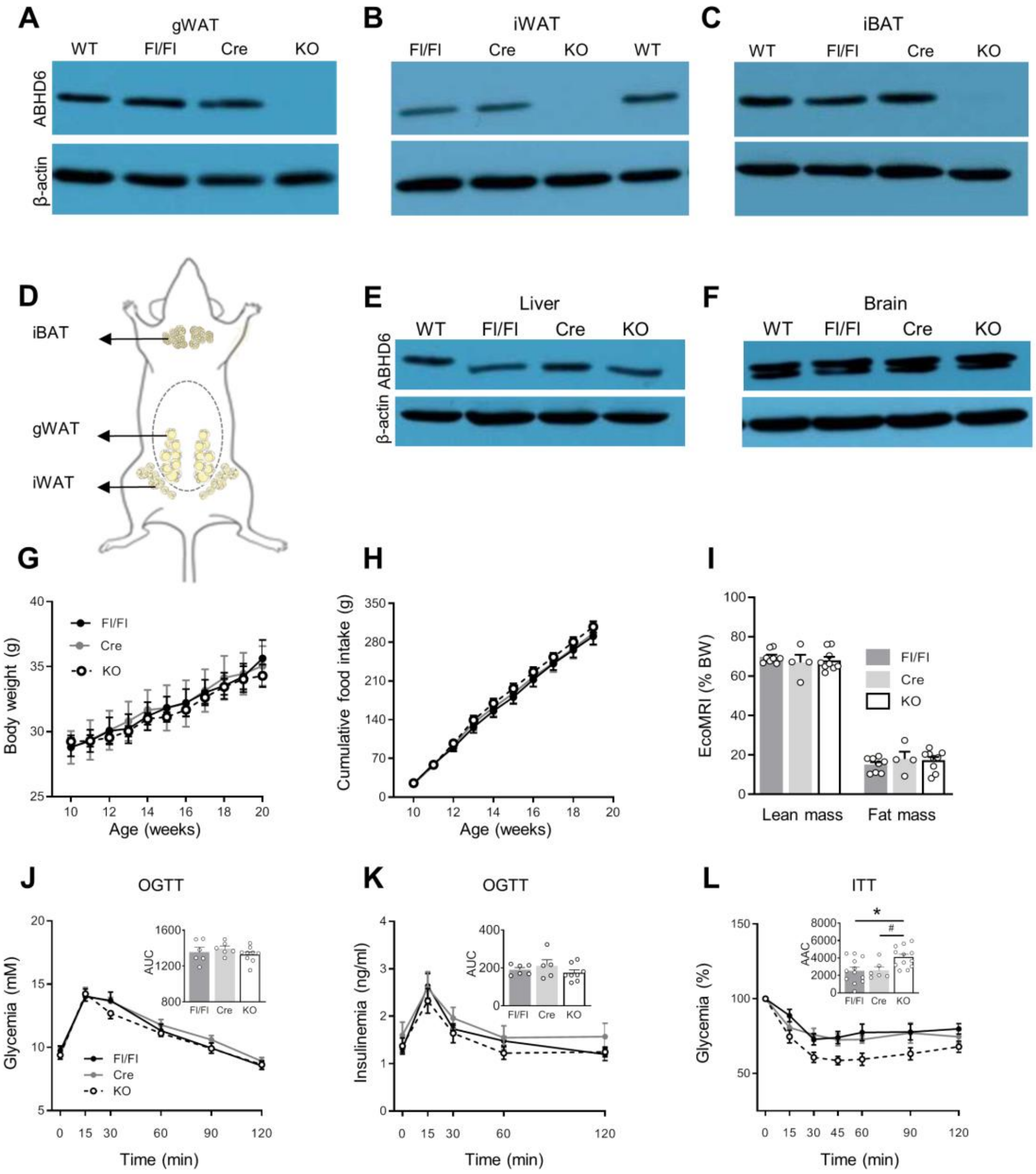

Supplemental Figure 1. Characterization of AT-ABHD6-KO mice at room temperature.

AT-ABHD6-KO mice were generated as detailed in the Methods. After tamoxifen treatments, mice were fed a standard chow diet for 12 weeks. Study groups consisted of *Abhd6*$^{flox/flox}$ (Fl/Fl), *Adipoq-Cre*/ERT2 (Cre), and AT-ABHD6-KO (KO). Oral glucose tolerance test (OGTT) was performed at 17 weeks of age, after a 6 h food deprivation. Insulin tolerance test (ITT) was performed on 20 weeks old mice, after a 4 h food

deprivation (5-13 mice/group). At the end of the feeding period, animals were analyzed by Echo-MRI, and then were sacrificed.
(A-C) ABHD6 protein level in gWAT, iWAT, and iBAT.
(D) Simplified schematic showing different fat depots in mice. Visceral perigonadal WAT (gWAT), subcutaneous inguinal WAT (iWAT), and interscapular BAT (iBAT).
(E and **F)** ABHD6 protein level in liver and brain.
(G) Body weight.
(H) Cumulative food intake.
(I) Lean and fat mass, expressed as percentage of body weight (BW).
(J and **K)** Glycemia and insulinemia during OGTT. Inset depicts area under the curve (AUC).
(L) Glycemia during ITT. Inset depicts area above the curve (AAC).
One-way ANOVA and Tukey post hoc test; Fl/Fl vs. KO: $^*p < 0.05$; Cre vs. KO: #$p < 0.05$.

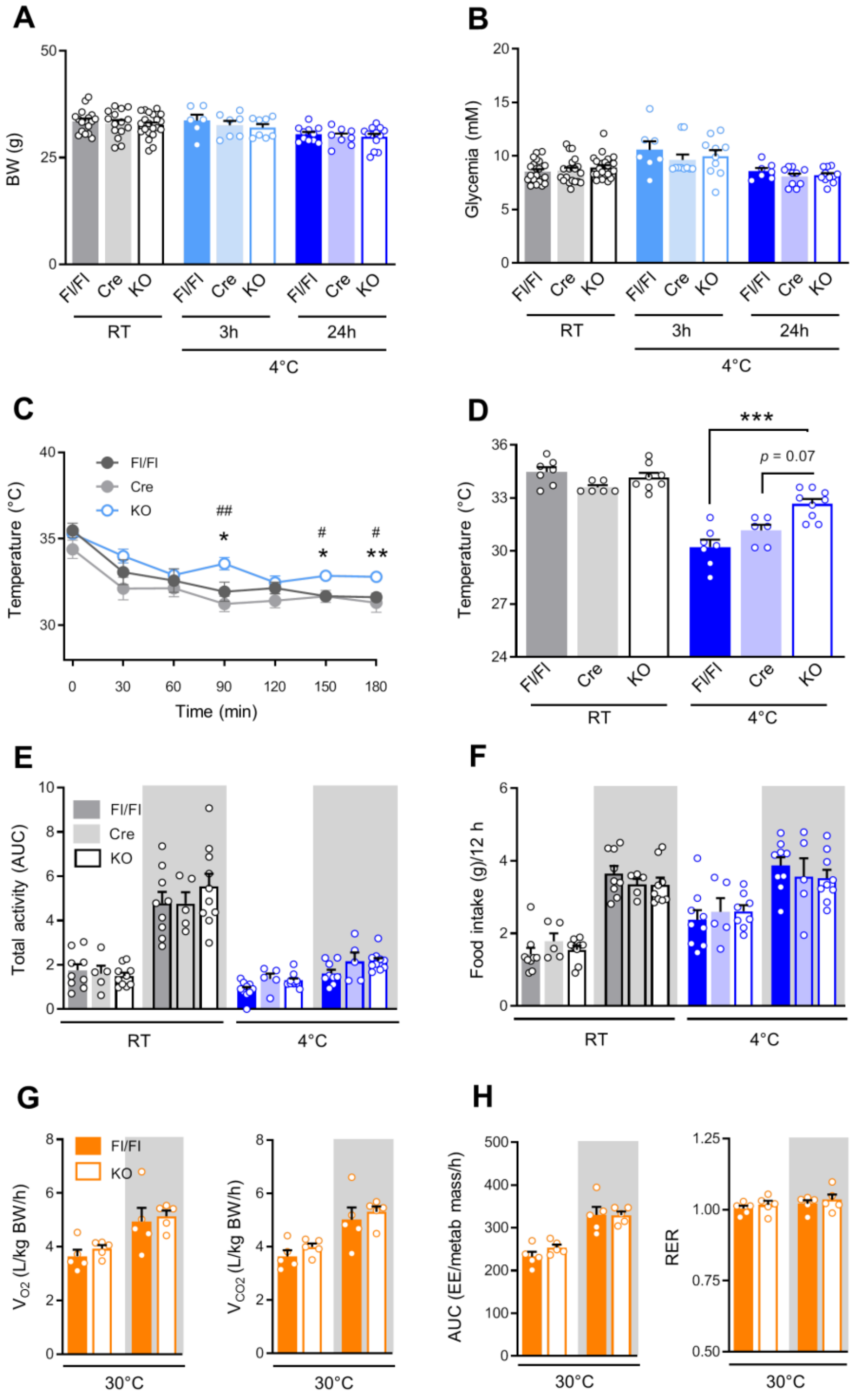

Supplemental Figure 2. Hypothermia resistance of AT-ABHD6-KO mice vs. controls, independent of total activity and food intake.

Mice were exposed to 4°C (3 h or 24 h, as indicated), maintained at RT, or housed at thermoneutrality (30ºC) for 5 days, as indicated.

(A) Body weight (BW) before and after cold-induction.
(B) Glycemia before and after cold-induction.
(C) Rectal temperature during 3 h cold-induction.
(D) Rectal temperature before and after 24 h cold-induction.
(E and **F)** Total activity and food intake data obtained from metabolic cages.
(G) Volume O_2 (V_{O2}) and Volume CO_2 (Vc_{O2}).
(H) Energy expenditure (EE; kcal/kg metabolic mass/h) and respiratory exchange ratio (RER).

Minimum of 5-9 mice/group; one-way ANOVA and Tukey post hoc test. Fl/Fl vs. KO: *$p < 0.05$, **$p < 0.01$, ***$p < 0.001$; Cre vs. KO: #$p < 0.05$, ##$p < 0.01$.

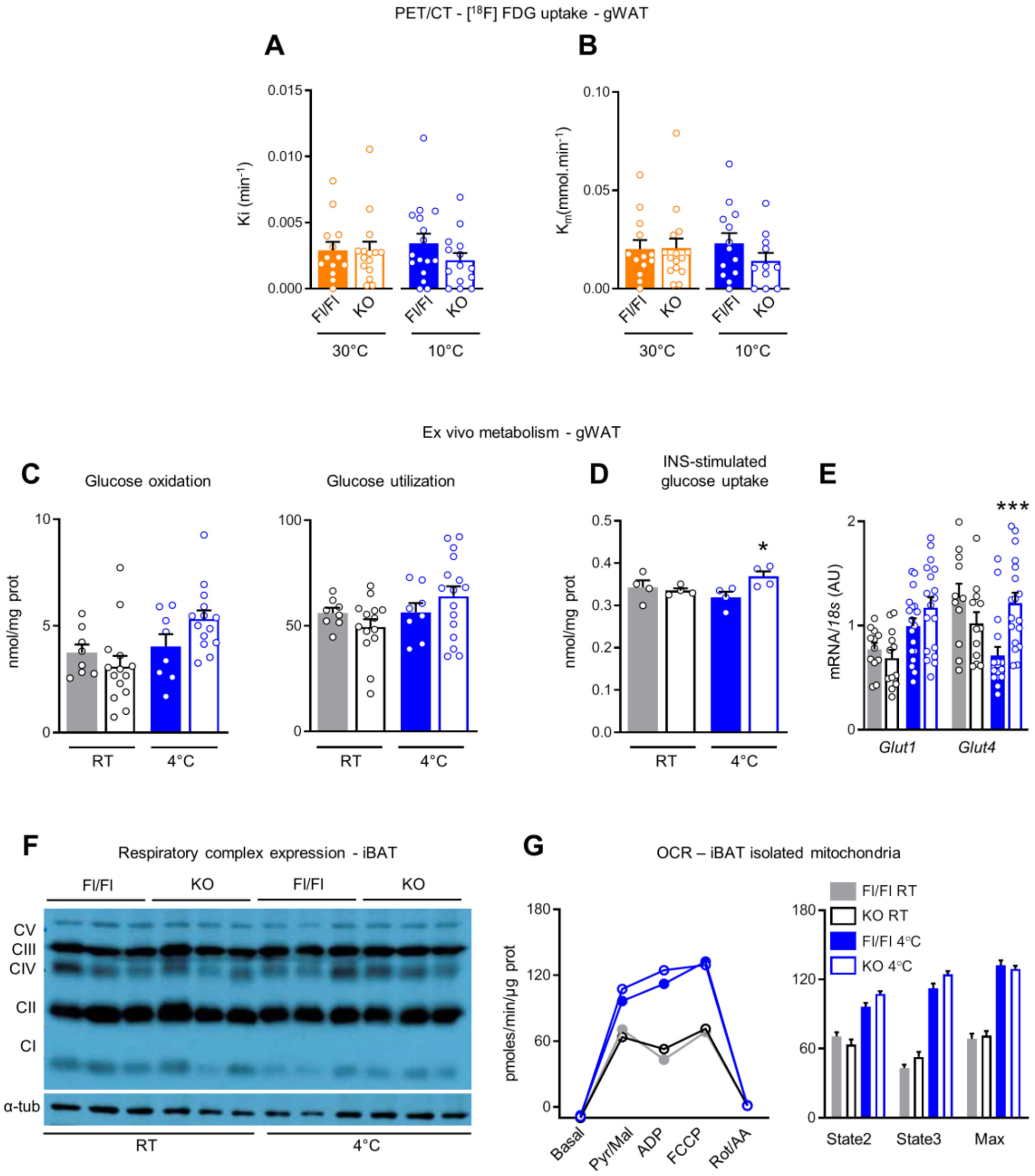

Supplemental Figure 3. ABHD6-deficient gWAT exhibited cold-dependent insulin-stimulated glucose uptake.

For in vivo PET/CT assays, mice were randomly placed at 30°C or 10°C for 48 h following by injection of the tracer. For ex vivo experiments mice were either exposed to 4°C for 24 h or kept at RT.

(A and B) Fractional (Ki) and net (Km) [^{18}F] FDG uptake by PET/CT at 30°C and 10°C (gWAT).

(C) Ex vivo glucose oxidation (^{14}C-glucose) and utilization (^{3}H-glucose).

(D) Ex vivo insulin-stimulated glucose uptake, using radiolabeled 2DG.

(E) Expression of glucose transporters.

(F) Representative Western blot measuring Complex I subunit NDUFB8, Complex II (succinate dehydrogenase B), Complex III (UQCRC2), Complex IV (cytochrome c oxidase subunit 1), Complex V (alpha-F1-ATP synthase), and tubulin in the total lysates of iBAT.

(G) Quantification of oxygen consumption rates (OCR) in isolated mitochondria from iBAT under the different respiratory states: pyruvate + malate (state 2), ADP (state 3), FCCP (maximal), and rotenone + antimycin.

(A and B) 5-15 mice/group; (C) 8-14 mice/group; (D) 4 mice/group; (E) 12-18 mice/group; (G) 6 mice/group. (A and B) Student's t-test; (C-E) one-way ANOVA and Tukey post hoc test. The effects of genotype: *$p < 0.05$, ***$p < 0.001$.

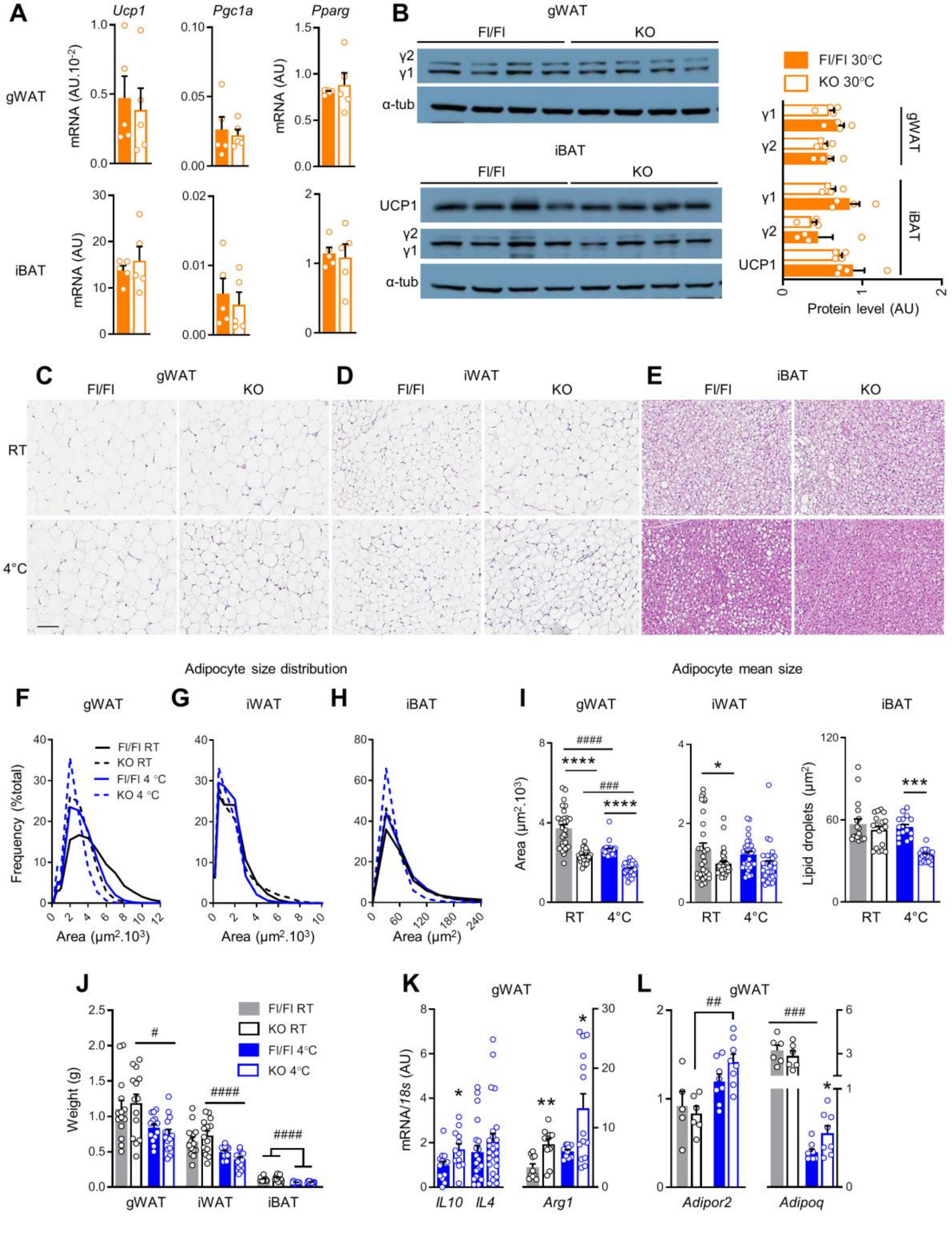

Supplemental Figure 4. Thermogenic markers express similarly in fat depots of two genotypes under thermoneutral condition, and smaller adipocytes and enhanced anti-inflammatory markers in gWAT of cold-exposed AT-ABHD6-KO mice.

Mice were exposed to 4°C for 24 h, maintained at RT, or housed at 30ºC for five days prior to sacrifice and adipose tissue collection.

(A) *Ucp1*, *Pgc1a* and *Pparg* mRNA expression.

(B) Protein level of PPARγ1 (γ1), PPARγ2 (γ2), UCP1, with α-tubulin (α-tub) as loading control.

(C-E) Representative images from hematoxylin and eosin (H&E) stained sections of adipose depots. Scale bar = 100 μm. Top panels show adipocyte morphology at RT and the bottom panels at 4°C.

(F-H) The frequency distribution of adipocyte cell/lipid droplet size, expressed as percentage.

(I) Mean adipocyte/lipid droplet area (μm^2).

(J) Adipose tissue weight (g).

(K and **L)** Expression of anti-inflammatory/insulin sensitivity marker genes in gWAT, normalized to *18s*.

(A) 5 mice/group; (C-I) 6 mice/group; from each mice 5 WAT sections and 3 BAT sections were analyzed; (I) 14 mice/group; (K and L) 5-24 mice/group. One-way ANOVA and Tukey post hoc test; the effects of genotype: *$p < 0.05$, **$p < 0.01$, ***$p < 0.001$, ****$p < 0.0001$; the effects of cold-induction: #$p < 0.05$, ##$p < 0.01$, ###$p < 0.001$, ####$p < 0.0001$.

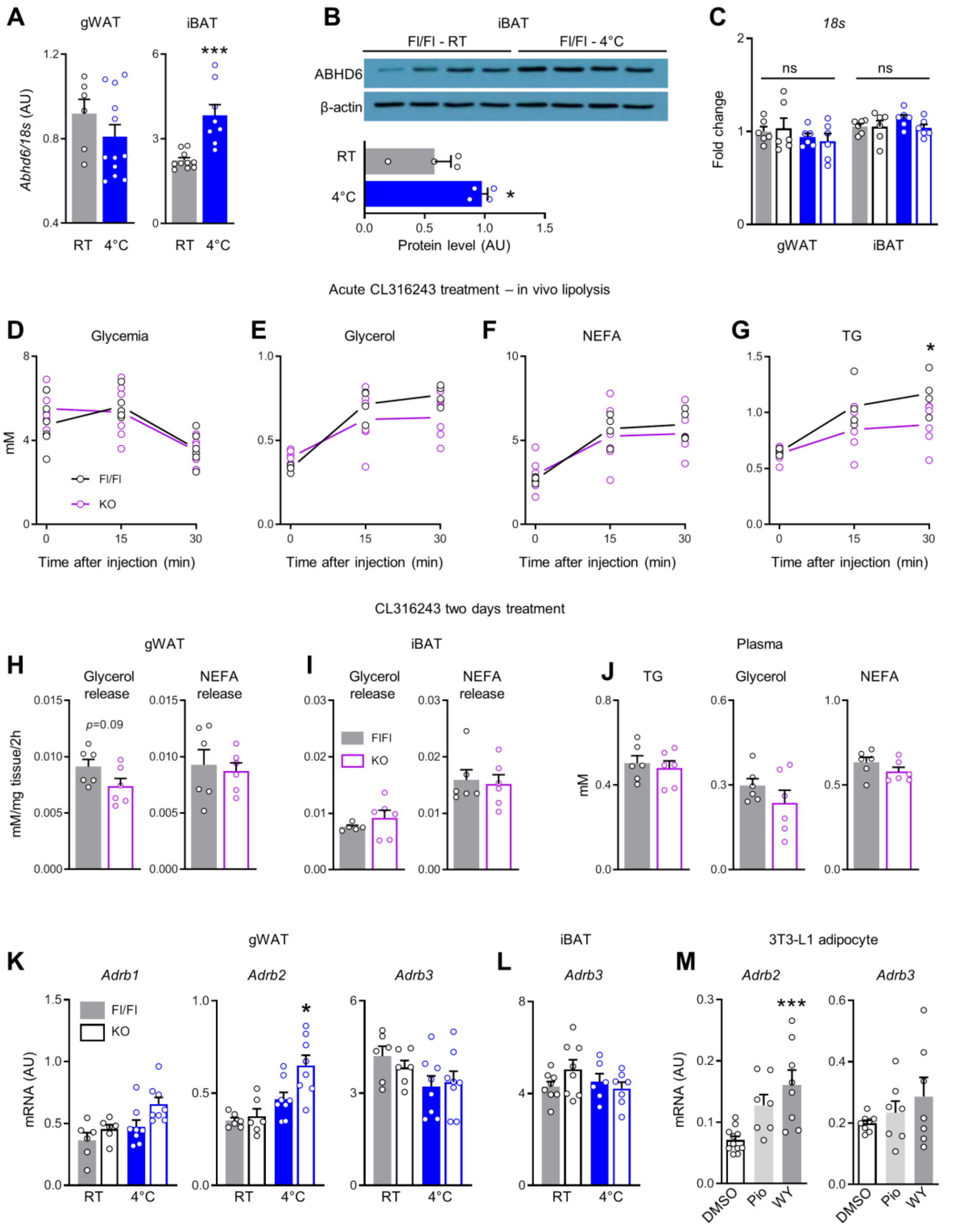

Supplemental Figure 5. Adipose-specific deletion of ABHD6 does not alter β-AR3-mediated lipolysis.

For cold exposure assay, mice were either exposed to 4°C or maintained at RT for 24 h. For CL316243 treatment, mice were injected with CL316243 (1 mg/kg/day) for two days,

prior to sacrifice and tissue/blood collection. For the CL316243 acute studies, mice were fasted overnight and blood collected within 30 minutes of the injection.
(**A**) *Abhd6* mRNA expression in Fl/Fl gWAT and iBAT at RT vs. cold.
(**B**) ABHD6 protein expression in Fl/Fl iBAT at RT vs. cold.
(C) *18s* mRNA expression in gWAT and iBAT at RT vs. cold.
(**D-G**) Glycemia and plasma glycerol, NEFA and TG levels upon acute CL316243 treatment.
(**H** and **I**) Ex vivo measurement of glycerol and NEFA released from gWAT and iBAT tissue explants after two days of CL316243 treatment.
(**J**) Plasma glycerol, NEFA and TG levels after two days of CL316243 treatment.
(**K**) *Adrb1, Adrb2 and Adrb3* mRNA expression in gWAT.
(**L**) *Adrb3* mRNA expression in iBAT.
(**M**) Expression of *Adrb2* and *Adrb3* was measured 24 h after treatment of 3T3-L1 adipocytes with 50 μM WY-14643 (WY) and pioglitazone (Pio).
(A and D-M) min 6 mice/group; (A and B, and H-J) Student's t-test; (D-G) Two-way ANOVA; (K-M) One-way ANOVA and Tukey post hoc test; $*p < 0.05$, $***p < 0.001$.

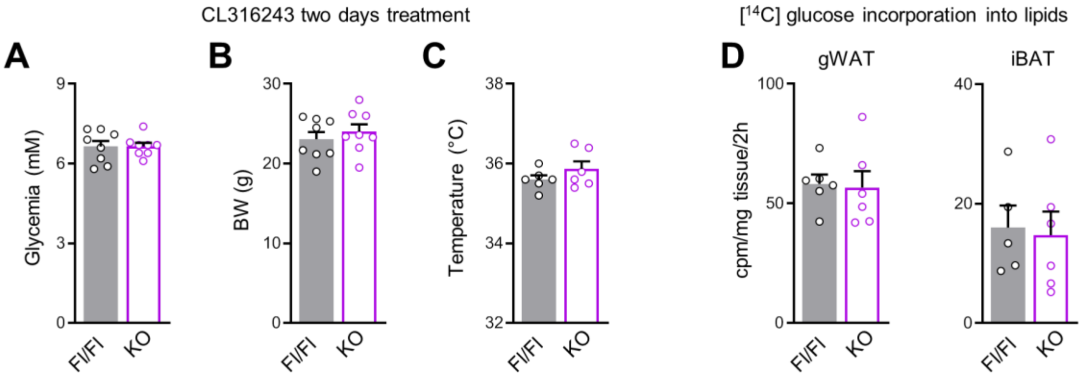

Supplemental Figure 6. Metabolic features of CL316243-treated mice and ex vivo gWAT and iBAT de novo lipogenesis.

10-12 weeks old AT-ABHD6-KO and Fl/Fl mice were injected with CL316243 (1 mg/kg/day) for two days, prior to sacrifice and tissue/blood collection (6-8 mice/group).

(A- C) Glycemia, body weight (BW) and rectal temperature.

(D) Ex vivo de novo lipogenesis (^{14}C-glucose).

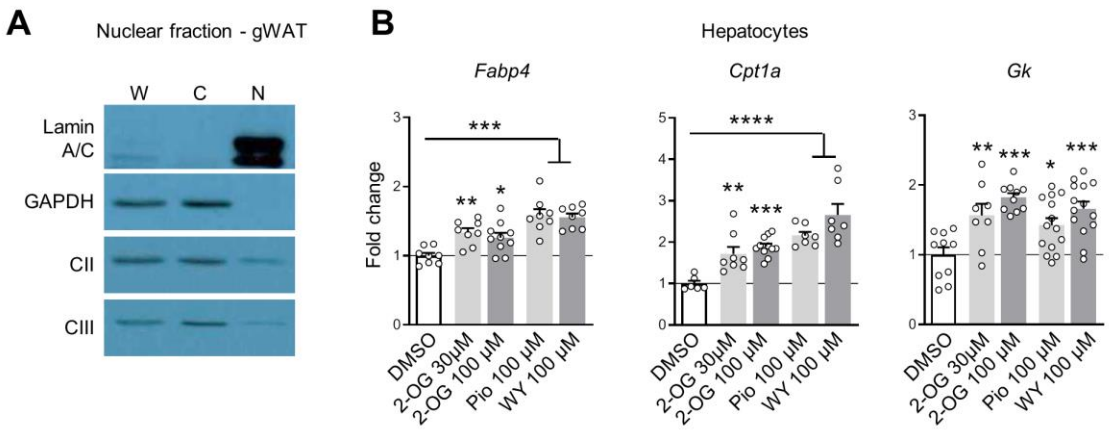

Supplemental Figure 7.

(A) Validation of nuclear fraction isolated from gWAT. W: whole lysate; C: post nuclear fraction; N: nuclear fraction.

(B) Expression of PPARα and PPARγ target genes (*Fabp4*, *Cpt1a* and *Gk*) were measured 24 h after treatment of primary hepatocytes with 2-oleoylglycerol (2-OG), using WY-14643 (WY) and pioglitazone (Pio) as positive controls. Two independent experiments; minimum 6 wells/condition. One-way ANOVA and Tukey post hoc test: *$p < 0.05$, **$p < 0.01$, ***$p < 0.001$, ****$p < 0.0001$.

Supplemental Table 1. Study models and chemical/biological materials.

	SOURCE	REFERENCE/CATALOGUE #
Antibodies		
Anti-ABHD6	Gift from Dr. J. Mark Brown, Lerner Research Institute, Cleveland, OH (Gwynneth T. et al., Cell Rep, 2013)	N/A
Anti-ABHD6	Cell Signaling Technology	cat: 97573; clone: D3C8N
Alexa Fluor 488 conjugated secondary antibody	Thermo Fisher Scientific	cat: A21206
Anti-β-actin	Sigma-Aldrich	cat: A5441; clone: AC-15
Anti-UCP1	Abcam	cat: ab10983
Anti-α-tubulin	Abcam	cat: ab4074
Anti-PGC-1α Mouse mAb	Sigma-Aldrich	cat: ST1202; clone: 4C1.3
Total OXPHOS antibody	Abcam	cat: ab110413
Anti-ATGL Rabbit mAb	Cell Signaling Technology	cat: 2439; clone: 30A4
Anti-ABHD5 Polyclonal	Abnova Corporation	cat: PAB8649
Anti-phospho-HSL (Ser563)	Cell Signaling Technology	cat: 4139
Anti-HSL	Cell Signaling Technology	cat: 4107
Anti-MAGL Rabbit Polyclonal	Abcam	cat: ab24701
Anti-GAPDH Rabbit mAb	Cell Signaling Technology	cat: 2118; clone: 14C10
Anti-Lamin A/C Mouse mAb	Cell Signaling Technology	cat: 4777S; clone: 4C11
Chemicals		
Insulin	Sigma-Aldrich	cat: I6634
IBMX	Sigma-Aldrich	cat: I5879
Dexamethasone	Sigma-Aldrich	cat: D2915
Oil Red O	Sigma-Aldrich	cat: O0625
Collagenase type II	Sigma-Aldrich	cat: C6885
FA–free BSA	Sigma-Aldrich	cat: A6003
Isoproterenol	Sigma-Aldrich	cat: I6504
CL316243	Sigma-Aldrich	cat: 5976
Pioglitazone	Toronto Research Chemicals	cat: P471000
WY14643	Enzo Life Sciences	cat: BML-GR200
2-Oleoylglycerol	Sigma-Aldrich	cat: M2787
Antimycin-A	Sigma-Aldrich	cat: A-8674
Rotenone	Sigma-Aldrich	cat: R8875
ADP	Sigma-Aldrich	cat: A-5285
FCCP	Sigma-Aldrich	cat: C2920
SYBR Green	QuantiTect	cat: 204145
DMEM, high glucose	Thermo Fisher Scientific	cat: 11965
Lipofectamine 2000	Thermo Fisher Scientific	cat: 11668
Commercial Kits		
HR Series NEFA Color Reagent A	Wako Diagnostics	cat: 999-34691
HR Series NEFA-HR Solvent A	Wako Diagnostics	cat: 995-34791
HR Series NEFA Color Reagent B	Wako Diagnostics	cat: 991-34891
HR Series NEFA-HR Solvent B	Wako Diagnostics	cat: 993-35191
Triglyceride reagent	Sigma-Aldrich	cat: T2449
Free glycerol reagent	Sigma-Aldrich	cat: F6428
Insulin ELISA kit	ALPCO	cat: 80-INSMS-E10
Luciferase assay kit	Promega	cat: E1501

**Cell Lines**		
HEK293T	ATCC	ATCC CRL-1573
3T3-L1	ATCC	ATCC CL-173
**Mouse Models**		
Mouse: *Abhd6*$^{flox/flox}$	Zhao et al., Cell Metab, 2014	N/A
Mouse: *Adipoq-Cre*/ERT2	Gift from Dr. Stefan Offermanns, Goethe University, Frankfurt, Germany (Sassmann et al., Genesis, 2010)	N/A
**Oligonucleotides**		
RT-qPCR Primers	This paper	See Supplemental Table 2
**Recombinant DNA**		
PPARα	ORIGENE	cat: SC308984
PPARγ	ORIGENE	cat: SC124236
PPRE-X3-TK-luc	Addgene	cat: 1015
**Software**		
GraphPad Prism 6	GraphPad Software	https://www.graphpad.com/
ImageJ Version 1.48v	NIH, MD, USA	https://imagej.nih.gov/ij/
AMIDE-bin 1.0.5	Crump Institute for Molecular Imaging, UCLA, USA	http://amide.sourceforge.net
**Radioactive Tracers**		
Deoxy-D-glucose, 2-[1,2-3H(N)]	Perkin Elmer	cat: NET549001MC
D-[14C(U)]-Glucose	Perkin Elmer	cat: NEC042X001MC
2-[18F]fluoro-2-deoxy-glucose tracer	Synthetized at Sherbrooke cyclotron	N/A
[11C]-acetate tracer	Synthetized at Sherbrooke cyclotron	N/A

Supplemental Table 2. **Primer sequences used for RT-PCR.**

Gene		Primer Sequence
m18s	Forward	CTG AGA AAC GGC TAG CAC ATC
	Reverse	GGC CTC GAA AGA GTC CTG TAT
mAbhd6	Forward	AGA CCA GGT GCT TGA TGT
	Reverse	CTC TCC ATC ACT ACC GAA T
mHsl	Forward	GGC TCA CAG TTA CCA TCT CAC C
	Reverse	GAG TAC CTT GCT GTC CTG TCC
mAtgl	Forward	TCC CAC TTT AGC TCC AAG GAT
	Reverse	AGC TTC CTC TGC ATC CTC TTC
mMgll	Forward	GTG CCT ACC TGC TCA TGG AAT
	Reverse	GAG GAC GGA GTT GGT CAC TTC
mPparg	Forward	GGT CAG CTC TTG TGA ATG GAA
	Reverse	ATC AGC TCT GTG GAC CTC TCC
mPpara	Forward	GGC CAT ACA CAA GGT CTC CAT
	Reverse	AGA GAA TCC ACG AAG CCT ACC
mUcp1	Forward	CTT TGC CTC ACT CAG GAT TGG
	Reverse	ACT GCC ACA CCT CCA GTC ATT
mPgc1a	Forward	TAG AGT GTG CTG CTC TGG TTG
	Reverse	GAT TGG TCG CTA CAC CAC TTC
mArg1	Forward	AGA CCA CAG TCT GGC AGT TG
	Reverse	CCA CCC AAA TGA CAC ATA GG
mIl4	Forward	GGT CTC AAC CCC CAG CTA GT
	Reverse	GCC GAT GAT CTC TCT CAA GTG AT
mIl10	Forward	CTT ACT GAC TGG CAT GAG GAT CA
	Reverse	GCA GCT CTA GGA GCA TGT GG
mAdipoq	Forward	GTG TGT TCC TGC TTC ATT CC
	Reverse	TGG TCA GAA GTT GGA GGT TC
mAdipoR2	Forward	CCTTTCGGGCCTGTTTTAAGA
	Reverse	GAGTGGCAGTACACCGTGTG
mGlut1	Forward	GCT GTG CTT ATG GGG TTC TC
	Reverse	CAC ATA CAT GGG CAC AAA GC
mGlut4	Forward	ACA TAC CTG ACA GGG CAA GG
	Reverse	GCG CCT TAG TTG GTC AGA AG
mGpam (aka, GPAT1)	Forward	CGG AAC TGA ACT GGA GAA GTG
	Reverse	GAT GAA TTG CTG GTG CTC CTT
mGpat3	Forward	CCT TGG AGT GGG CCA CAT TA
	Reverse	AGA GCT CGA AGT CCC TTC CT
mDgat1	Forward	GAG CTA TCC AGA CAA CCT GAC C
	Reverse	AGC ATC TCA AGA ACT CGT CGT
mGk	Forward	CAA ATG CAA GCA GGA CGA TG
	Reverse	GGC CCC AGC TTT CAT TAG G
mLpp3	Forward	CCA TCC TGG CGA TCA TTA CAG
	Reverse	AAA GGA AGC ATC CCA CTT GCT
mLpin1	Forward	AAG AGA CTG ACA ACG ATC AGG A
	Reverse	TTC CCC AGA GAA CCA GTG GAT
mAgpat2 (aka, LPAAT)	Forward	TCA CCT CAG GAA CAA TCA AGG
	Reverse	AAT GGC AGA GTT CTC TTG GG
mDgkd	Forward	GCC GAC AAC AGG AAA GAA ATG
	Reverse	TTG GGT AGG CTC AAA ATG CTC
mAgk	Forward	CTC ATT CCT CCC AAT GCA CAA
	Reverse	TCC ATG CCA GAC AGG TGT AAA
mAdrb1	Forward	CAT CAT GGG TGT GTT CAC G
	Reverse	GAA GAC GAA GAG GCG ATC C
mAdrb2	Forward	TTG CAG TGG ATC GCT ATG TTG
	Reverse	TGA CCA CTC GGG CCT TAT TCT
mAdrb3	Forward	ACT GCT AGC ATC GAG ACC TTG
	Reverse	AAG GGT TGG TGA CAG CTA GG
mAqp7	Forward	GTG CCA TTA ACC ACT TTG CAG
	Reverse	CGG TAA CAA GGA TGC CTA TCA
mFabp4 (aka, Ap2)	Forward	CCA TCT AGG GTT ATG ATG CTC TTC
	Reverse	ACA CCG AGA TTT CCT TCA AAC TG
mCpt1a	Forward	GGT TCA AGC TGT TCA AGA TAG C
	Reverse	ACC ACA TAG AGG CAG AAG AGG
